# Dental-craniofacial manifestation and treatment of rare diseases

**DOI:** 10.1038/s41368-018-0041-y

**Published:** 2019-02-20

**Authors:** En Luo, Hanghang Liu, Qiucheng Zhao, Bing Shi, Qianming Chen

**Affiliations:** State Key Laboratory of Oral Diseases & National Clinical Research Center for Oral Diseases, West China Hospital of Stomatology, Sichuan University, Chengdu, China

**Keywords:** Oral diseases, Oral manifestations

## Abstract

Rare diseases are usually genetic, chronic and incurable disorders with a relatively low incidence. Developments in the diagnosis and management of rare diseases have been relatively slow due to a lack of sufficient profit motivation and market to attract research by companies. However, due to the attention of government and society as well as economic development, rare diseases have been gradually become an increasing concern. As several dental-craniofacial manifestations are associated with rare diseases, we summarize them in this study to help dentists and oral maxillofacial surgeons provide an early diagnosis and subsequent management for patients with these rare diseases.

## Oral Diseases: Dental and craniofacial indications associated with rare diseases

Rare diseases are typically genetic, chronic, incurable disorders with a relatively low incidence, and a number of dental and craniofacial manifestations are associated with such diseases. A team headed by En Luo and Hanghang Liu at Sichuan University in China conducted a large-scale review of the manifestations and treatment of rare diseases related to dental and craniofacial disorders. The authors found that in their early stages, several rare diseases exhibit distinctive dental and craniofacial characteristics. Provided they understand the clinical features, dentists and oral surgeons are the individuals best placed to make an early identification of such diseases. The authors believe that their findings will help clinicians fully grasp the related clinical signs and potential treatments of rare diseases, thereby facilitating early diagnosis and patient management as well as improving prognosis.

## Introduction

Recently, the National Health and Health Committee of China first defined 121 rare diseases in the Chinese population. The list of these rare diseases was established according to prevalence, disease burden and social support, medical technology status, and the definition of rare diseases in relevant international institutions. Twenty million people in China were reported to suffer from these rare diseases.

A rare disease is any disease or condition that affects a small percentage of the population, and most of them are genetic and life-threatening diseases.^1,2^ Most rare diseases appear early and throughout the person’s life, and 30% of those affected will die before 5 years of age.^3^ However, there has been no single, widely accepted definition for rare diseases until now. According to national conditions, each country or region may have different criteria for rare disease identification. In the United States, a rare disease is defined by the Rare Diseases Act of 2002, which relies solely on prevalence: 'any disease or condition that affects fewer than 200 000 people in the United States', or ~1 in 1 500 people.^4^ In the European Union, the European Commission defines rare disease as a life-threatening or chronically debilitating disease with a population prevalence of less than 1 in 2 000.^4^

In several parts of the world, rare disease is used as a synonym of ‘orphan disease’, indicating a lack of a sufficiently large market to obtain source and support for discovering and investigating-related therapies. Paradoxically, rare diseases are common.^5^ More than 7 000 rare diseases, approximately 10% of the total human diseases, have been identified with advances in our knowledge regarding the human genome, and more than 2 000 million people worldwide are living with one of the 7 000 diseases defined as “rare”.^6^ In recent years, several rare diseases have been gaining a large amount of attention, such as amyotrophic lateral sclerosis. Increased concern and a correct understanding of rare diseases would promote mechanistic, diagnostic and therapeutic advances.

In this review, we aim to summarize the related manifestations and treatment of dental-craniofacial disorders related to rare diseases, thus helping to improve understanding and certainly diagnostic capacity for dentists and oral maxillofacial surgeons.

## Dental-craniofacial disorder-related rare diseases

### Tooth dysplasia

#### Congenital ectodermal dysplasia

Ectodermal dysplasias (EDs) are a group of more than 150 different genetic disorders deriving from ectodermal structural abnormalities.^7,8^ Ectodermal dysplasias have been described as 'heritable conditions in which there are abnormalities of two or more ectodermal structures, such as the hair, teeth, nails, sweat glands, salivary glands, cranial-facial structure, digits and other parts of the body'.^7^ The abnormality in the development of tooth buds frequently results in congenital hypodontia (both primary and permanent dentitions) and/or changes in tooth morphology or size, such as peg-shaped or pointed teeth, taurodontism and enamel defects, including hypoplasia.^9^ The degree of tooth missing is always in the mild to moderate range, and a wide variation is observed regarding which teeth are missing; however, the most frequently reported missing teeth are the first molars, upper central incisors and canines^10–14^ (Table 1). Accordingly, composite restorations or crowns are almost always necessary for children as early as 2 years of age, and multiple denture replacements are often needed as the child grows, with dental implants providing a potential option in adolescence when the jaw is fully grown. The current option of extracting teeth and substituting them with dental implants is quite common.^15^ Additionally, orthodontic treatment is further necessary during the early teenage years as part of the best multi-disciplinary approach. Furthermore, several studies have also reported reduced salivary secretion in ED patients, accompanied by a reduced buffering ability and increased bacterial counts.^16–20^ Therefore, a systematic preventive plan including fluoride use and the placement of fissure sealants will be especially important for patients with ED owing to their increased caries risk.

**Table 1:** Dental-craniofacial manifestations of tooth dysplasia-related rare diseases

Rare diseases	Aetiology	Major manifestations	Dental-cranio-facial manifestations	Incidence^a^	Onset period^b^
Congenital ectodermal dysplasia	Abnormalities of the ectodermal structures	Excessively fragile and twisted hair; thick, brittle or discoloured nails; red or brown pigmentations on skin; overheating; respiratory infections	Hypodontia, peg-shaped or pointed teeth, taurodontism, enamel hypoplasia; Reduced salivary secretion	80%; 30.2%	Childhood Childhood
Williams syndrome	Genetic deletion of chromosome 7q11.23	Intellectual disability; cardiovascular defects; failure to thrive; lack of social inhibition	Small jaw, wide mouth with full lips; Malocclusions with widely spaced teeth, hypodontia and enamel defects	75%–91% 38%–93%	Childhood Early childhood
Congenital erythropoietic porphyria	Genetic mutations in chromosome 10q25.2-q26.3	Thickened skin with hyperpigmentation and bullae formation; anaemia; dry eyes	Facial scabs and scars; Teeth: reddish fluorescence, reddish-brown discoloration with a sharply defined margin; Sclerotic and osteolytic round lesions in the skull, maxilla, mandible	47% 73% 41%	Early childhood Childhood Adulthood

^a^Incidence of dental-craniofacial manifestations

^b^Onset period of dental-craniofacial manifestations

#### Williams syndrome

Williams syndrome (WS, also known as Williams–Beuren syndrome) is a multi-system genetic disorder that is caused by a genetic abnormality, especially the deletion of a specific region in chromosome 7q11.23 containing 26–28 genes, such as *CLIP2*, *ELN, GTF2I, GTF2IRD1, BCL7B* and *LIMK1*.^21–25^ Most of the gene deletions occur as a random event during the embryonic period rather than being inherited from affected parents, and different clinical features are believed to be linked to the loss of specific genes.^26^

WS is characterized by various degrees of intellectual disability or mental retardation, unique facial features, cardiovascular defects and hypersocial behaviour, and cardiovascular complications are the major cause of death in WS patients. Regarding physical disorders, ophthalmologic and auditory abnormalities (altered visual acuity, strabismus, sensorineural hearing loss and hyperacusis), cardiovascular diseases (vascular stenosis, hypertension and stroke), gastrointestinal diseases (constipation, colic, rectal prolapse and coeliac disease) and musculoskeletal disorders (joint laxity, joint contractures and scoliosis) are always observed in WS patients.^21,27–29^ Additionally, WS patients usually exhibit distinctive facial features, including a broad forehead, bitemporal narrowing, a short nose with a broad tip, malar flattening, full cheeks, a small jaw, a wide mouth with full lips and large ear lobes, as well as dental abnormalities, such as small or unusually shaped primary teeth, malocclusions with widely spaced teeth, hypodontia and tooth enamel defects.^21,30–32^ (Table 1) Regarding the nervous system, multiple missing genes result in several defects in the cerebellum, right parietal lobe, and left frontal cortical regions. Thus, patients with WS often present a cognitive impairment, poor balance and coordination, defects in language development and coordination of fine motor tasks, such as drawing and writing, and disabilities in visuospatial construction.^21,33–37^ Furthermore, WS patients consistently demonstrate an overly friendly, highly social and empathic personality, as well as excessive worry and fears, distractibility, and irritability. However, these patients have been reported to show a greater volume of memory and higher rhythm propensity and fondness of music.^38–40^ Concerning endocrine abnormalities, hypercalcemia, diabetes mellitus, subclinical hypothyroidism and other endocrine disturbances are well described in some patients with WS, which might lead to muscle hypotonia, a diminished appetite, obesity and a below-average height and weight.^21,38,41–43^

Current management guidelines for WS have not been established, and the available treatments involve a combination of medical monitoring, pharmacotherapy, surgery, speech and behavioural treatments. Routine supervision of blood pressure, blood glucose and calcium levels are generally recommended for all patients with WS, surgery or stent insertion is the preferred method for moderate to severe aortic stenoses, an oral hypoglycaemic agent or insulin administration might be required for WS patients with potential diabetes mellitus, and bisphosphonate therapy is used for WS patients with significantly decreased bone density.^21,44–46^ Additionally, the application of anxiolytic and antipsychotic agents is occasionally prescribed, and behavioural treatments, such as the practice of relaxation, music treatment and social skill training, might be effective to channel the nature of affected patients.^47^ Other treatments, including dental restoration and orthodontic treatments, are administered depending on the patient's particular symptoms, and an early dental evaluation and dietary counselling are necessary to determine the presence of dental anomalies, such as caries and enamel structural defects.^32^

#### Congenital erythropoietic porphyria

Congenital erythropoietic porphyria (CEP), also known as Gunther’s disease, is an autosomal-recessive genetic disorder resulting from a homozygous defect in uroporphyfinogen III cosynthase, located on human chromosome 10q25.2-q26.3, which leads to the overproduction and accumulation of porphyrin I and coproporphyrins I.^48,49^ The accumulation of porphyrins first occurs in the bone marrow, followed by their release from circulating erythroid cells into plasma and deposition in tissues including bones, skin and brain.^50^ The phototoxic oxygen-dependent damage caused by electron transfer between excited porphyrins and the targets leads to a loss of membrane integrity, destruction of cellular organelles and cell apoptosis.^51^

Severe cutaneous photosensitivity due to massive porphyrin accumulation in the skin, characterized by subepidermal blistering with inflammatory infiltration, is the most frequently observed manifestation in CEP patients, although significant phenotypic variability in CEP has been reported.^52^ The severity of skin photosensitivity depends on the amount of porphyrin in the tissue,^53^ and thickened skin with hyperpigmentation as well as the rapid development of vesicles and bullae can be observed in any sun-exposed areas.^50^ Additionally, facial scabs and scars and the destruction of auricular and nasal cartilages, cheeks, lips and forehead can be severe due to repetitive skin damage and bone resorption, resulting in unique facial features.^54^ The accumulation of excessive porphyrin in erythroid precursors, reticulocytes and erythrocytes can induce osmotic haemolysis and subsequently result in mild to severe anaemia, and haematological complications are the major predictors of a poor prognosis for patients with CEP.^55^ Dental disorders are also found in most CEP patients due to porphyrin deposition during tooth development, which exhibits fluorescence under long-wavelength ultraviolet light as well as visible reddish-brown discoloration with a sharply defined margin^56^ (Table 1). Ocular complications, including corneal scarring, loss of eyelashes and eyebrows, ulcerative keratitis and conjunctivitis, and skeletal abnormalities including fractures and a shortened stature, are also frequently observed in CEP patients.^57–59^ Other symptoms, such as neurological manifestations, are not common, although Parkinson disease and corticobasal syndrome have been reported to be involved in some CEP patients.^60,61^

Multiple therapies have been proposed to treat CEP, such as the elimination of sun exposure using sunscreens (zinc oxide and titanium dioxide), oral β-carotene, or protective clothing; prevention of the reabsorbance of porphyrins with oral activated charcoal and cholestyramine; erythrocyte transfusions; and hematopoietic stem cell transplantation (HSCT); HSCT is the only curative method for severe CEP.^62–64^ Other management strategies, such as topical lubrication for dry eyes, glucocorticosteroid therapy for anaemia and thrombocytopenia and splenectomy for splenomegaly, have also been reported for specific complications.^65,66^

### Bone tissue abnormality

#### Osteogenesis imperfecta

Osteogenesis imperfecta (OI) is represented by a group of genetic disorders that mainly affect the bones, connective tissue and may increase skeletal fragility, also known as brittle bone disease.^67,68^ Among all cases, 85%–90% present a lack of type I collagen due to a mutation in the *COL1A1* and *COL1A2* genes that is inherited from the parents or develops de novo.^64^ With advances in genomic analysis and exome sequencing, several other gene mutations, such as *CRTAP, LEPRE1 PPIB, SERPINH1*, and *SP7* mutations, which might result in defects in collagen post-translational modifications or osteoblast differentiation, have been found to be involved in the occurrence of OI.^69^

OI had been recognized since the early 1980s. Fractures caused by mild trauma, bowing deformities of the long bones, and growth deficiency are the hallmark features, including macrocephaly and chest wall deformities. Additionally, typical extraskeletal manifestations can be associated variably with the disorder, including a dark or blue sclera, dentinogenesis imperfecta, pulmonary function impairment, the presence of wormian bones on skull radiographs, hyperlaxity of the ligaments and skin, and hearing impairment. Blue sclera and dentinogenesis imperfecta are always used as diagnostic signs of OI, and dentinogenesis imperfecta occurs more frequently in primary teeth than permanent teeth.^70^ (Table 2) Hearing loss is rare in the first 20 years of life, but half of patients aged more than 50 years report hearing loss. Radiological or histological examination can reveal generalized osteopenia and some combination of gracile ribs, long-bone bowing, and vertebral compression.^69^ Several clinical and genetic classifications have emerged to encompass the rare forms of osteogenesis imperfecta, beginning with David Sillence^71^ in the 1970s; however, they are associated with respective limitations. Additionally, in 2016, Forlino^69^ proposed a genetic-functional metabolic classification that is dependent on both the involved gene function and clinical features, updating several new types to classic Sillence types I–IV. The current classification of OI types is still debated.

**Table 2:** Dental-craniofacial manifestations of bone tissue abnormality-related rare diseases

Rare diseases	Aetiology	Major manifestations	Dental-cranio-facial manifestations	Incidence^a^	Onset period^b^
Osteogenesis imperfecta	Mutations in the *COL1A1* or *COL1A2* genes	Bones fracture easily; long bones deformity and small stature; loose joints; blue-grey colour of the sclera; loss of hearing	Facial deformities with high risk of fracture; Dentinogenesis imperfecta; Malocclusion and delayed tooth eruption	60% 28%–80% 60%–80%	Childhood or adulthood Childhood Early childhood
Hypophosphatemic rickets	Mutations in the phosphate-regulating endopeptidase gene	Disproportionate short stature; bone deformity; bone pain; hearing loss	Primary craniosynostosis; Recurrent abscesses with carious and trauma free teeth; Delayed tooth eruption, taurodontism	— 10.5%–64.7% 42.1%–85.7%	At birth Early childhood Childhood
Hypophosphatasia	Mutations in tissue non-specific alkaline phosphatase genes	Perinatal HPP: soft calvarium, deformed limbs, respiratory failure; Infantile hypophosphatasia: poor feeding, flail chest; Childhood hypophosphatasia: delayed walking, frequent fractures, open fontanels; Adult hypophosphatasia: painful feet, femoral pseudofractures, arthritis	Unossified calvarium with separated cranial sutures; Early loss of deciduous teeth, shell teeth, impaired dentinogenesis, permanent dentition caries	31%–40% 14%	At birth Childhood
Marfan syndrome	Mutations in *FBN1* gene	Arachnodactyly, disproportionately long, slender limbs with thin, ectopia lentis; weak wrists, long fingers and toes; undue fatigue, shortness of breath, cold arms, hands, and feet	Long narrow skull, high arched palate, mandibular and maxillary hypoplasia; Crowed teeth and overbite	63.6% —	Childhood Childhood
McCune–Albright syndrome	Mutation in the gene *GNAS*	Multiple bone fibrous dysplasia; café-au-lait skin pigmentation; endocrine diseases (Precocious puberty, testicular abnormalities and hyperthyroidism)	Facial asymmetry with expanding fibrous dysplasia lesion; Oral mucosal pigmentation; Dental malocclusion, dentin dysplasia, taurodontism and high caries index	62%–100% 70%–90% —	Early childhood (3.4 years old) At birth Childhood
Kallmann syndrome	Isolated Gonadotropin-Releasing Hormone Deficiency	Failure to start or fully complete puberty, primary amenorrhoea or lack of testicle development; Total lack of sense of smell, hearing loss	Cleft palate, hare lip, high-arched palate; Hypodontia, malformed teeth and other dental abnormalities	25%–30% 5%–10%	At birth Early childhood
Fanconi anaemia (FA)	Mutations in *FA* or *FA*-like genes	Bone marrow failure, acute myeloid leukaemia; skin hyperpigmentation; short stature, abnormal thumbs, absent radii	Microcephaly, triangular face; Head and neck cancers	51% 14%	Infanthood and Early childhood Adulthood

^a^Incidence of dental-craniofacial manifestations

^b^Onset period of dental-craniofacial manifestations

There is no cure for OI, and all management strategies are symptom-based or complication-based. Regarding the medical management of OI, a multidisciplinary team is necessary. Physiotherapy, hydrotherapy and rehabilitation exercises focus on strengthening the muscles, restricting joint range of motion and improving the patients’ living ability.^72,73^ Audiology examination should be carried out in childhood, as patients with severe disease with pure conductive or sensory loss might require hearing aids or cochlear implants.^74^ Oral health management, including oral hygiene introduction (regular brushing and flossing to avoid tooth chipping and potential fracture during dental procedures), and crown restoration should be applied to prevent caries, periodontitis and to optimize aesthetics. Regarding drug therapies, bisphosphonates are antiresorptive drugs that are widely used in children with OI to increase the volume of bone, bone strength, restore vertebral size and shape and decrease fractures, although the newly formed bones still contain defective collagen, and patients who use bisphosphonates should be well-informed of bisphosphonate-related osteonecrosis of the jaws.^69,75^ Concerning orthopaedic surgery, lower extremity, upper extremity and spinal surgery are usually conducted, and combined with pre- and post-surgical rehabilitation, placement of an intramedullary telescoping rod can be applied after correction of the bone deformity to provide strength, alignment and to stabilize fractures.^76^ Hip and knee arthroplasty are frequently conducted for OI patients with joint osteoarthritis,^77^ and spinal fusion is generally used to correct the scoliosis in OI patients.^78,79^ Because pulmonary impairment is the leading cause of death in OI patients, potentially as a secondary effect of scoliosis and rib fracture, treatment for pulmonary complications, such as obstructive disease, should also be emphasized to avoid respiratory infections and insufficiency.^80,81^

#### Hypophosphatemic rickets

Hypophosphatemic rickets is a genetic X-linked dominant form of rickets, also called X-linked hypophosphatemia (XLH), which is different from most cases of rickets in that the administration of vitamin D is not effective. XLH is caused by a loss-of-function mutation in the phosphate-regulating endopeptidase gene, X-linked (*PHEX*), and results in overactivity of fibroblast growth factor 23 (FGF23).^82^ The excess circulating FGF23 inhibits vitamin D 1α-hydroxylation and phosphate reabsorption by the kidneys, leading to hypophosphatemia and defective mineralization of the bones and thus facilitating rickets and osteomalacia.^83^

The main manifestations of XLH patients are a disproportionately short stature, reduced growth rate and bone deformities, such as coxa vara, tibial torsion and lower limb bowing, which occur in children before the fusion of the epiphysis.^84^ Adult patients can present symptoms of osteomalacia, including myopathy, bone pain, neurological complications and insufficiency fractures, due to enthesopathy and ectopic calcification.^85^ In addition, primary craniosynostosis has been reported to be present at or soon after birth,^86^ and mineralizing enthesopathy, osteoarthropathy and ossification of the spinal ligaments have also been reported during adulthood.^87,88^

Along with skeletal abnormalities, dental implications are consistently observed, and some of the main manifestations are recurrent abscesses or sinus tracts associated with carious and trauma-free teeth of the primary and permanent dentition because of the dentin defect (wide predentin zones and tubular defects) and microdefects in the enamel, especially in the anterior teeth.^89–96^ The hypoplasia of the enamel and the lack of fusion of calcospherites in dentin facilitates microbial penetration, leading to pulp infection, pulp necrosis and finally periapical periodontitis and abscesses.^97^ Other dental-related symptoms have also been reported, such as delayed tooth eruption,^84,89^ taurodontism (large pulp chambers, short roots, prominent pulp horns and a thin enamel layer),^97–100^ and a hypoplastic alveolar ridge^91,101^ (Table 2).

Early diagnosis and medical intervention for XLH is always associated with better therapeutic outcomes. The current standard treatment for XLH consists of activated vitamin D metabolites, oral inorganic phosphate salts and growth hormone supplementation as replacement therapy from the time of diagnosis until growth is complete, which has been shown to improve adult height, alleviate bowing and reduce the requirement for necessary surgeries.^82,84,102–105^ When patients reach adulthood, combined application of replacement treatments and orthopaedic surgery is strongly suggested in symptomatic patients who have bone pain, severe bowing, tibial torsion or insufficiency fractures, to recreate a normal anatomic and mechanical axis.^102^ Multiple osteotomies, and arthroplasty if necessary, are performed for deformed bones, and then the bone pieces are realigned in an improved position using external fixators or intramedullary nails to achieve a straightened configuration.^104,106^ The application of burosumab, a recombinant human IgG1 monoclonal antibody that targets FGF-23 and inhibits its activity, has been reported to increase renal tubular reabsorption of phosphate and thereby increase serum phosphate and serum 1,25(OH)_2_D in phase 1 and 2 clinical trials in both paediatric and adult patients with XLH.^107–109^ Moreover, diverse dental treatments have been suggested for XLH patients, as well as routine radiographic control of the entire dentition, topical fluoride varnish and fissure sealing, stainless steel crowns or permanent crown restoration, and early non-surgical root canal treatment or surgical resection for teeth associated with apical periodontitis.^95^ Teeth with severe periradicular abscesses might require extraction followed by implant restoration.

#### Hypophosphatasia

Hypophosphatasia (HPP) is a type of genetic bone metabolic disorder that results from a molecular defect in tissue non-specific alkaline phosphatase (TNSALP) genes, and a dominant effect of the mutated allele is usually suspected to be the cause of the disease.^110,111^ TNSALP is a membrane-bound glycosylated enzyme encoded by the *ALPL* gene, and its physiological function has been proposed to be involved in extracellular matrix mineralization, ATP hydrolysis and skeletal development.^112^ The absence and reduced activity of TNSALP would result in increasing extracellular PPi in the bone matrix, an inhibitor of hydroxyapatite formation, which is an important component of bone and lead to rickets and osteomalacia.^113^ In addition to hard tissues, such as bone and teeth, TNSALP is also essential for pyridoxal 5′-phosphate dephosphorylation and vitamin B6 production; thus, other organs, such as muscles, brain and liver, can also be affected in HPP patients.^114^

A small number of mutations are recurrently found, which result in a large number of compound heterozygous genotypes and a wide range of clinical symptoms. Based on the appearance of the first symptom, HPP is divided into several subtypes (during gestation or at birth: perinatal hypophosphatasia; before the first 6 months of life: infantile hypophosphatasia; onset ≥6 months to 18 years of age; childhood hypophosphatasia; after 18 years of age: adult hypophosphatasia), according to the classification proposed by Fraser et al.^115^ and Whyte et al.^116^ Additionally, odontohypophosphatasia refers to the phenotype when dental disease (including premature loss of deciduous teeth, especially the anterior teeth; large pulp chambers; impaired dentinogenesis; and rare enamel hypoplasia) is the only clinical abnormality, and no radiographic and histopathological evidence of rickets and osteomalacia can be observed^117^ (Table 2). Perinatal HPP is the most severe form of HPP, which is usually characterized by caput membranaceum and deformed limbs, periodic apnoea with cyanosis and bradycardia resulting from chest deformities and lung hypoplasia, myelophthisic anaemia and intracranial haemorrhage at birth.^118,119^ Nearly all the bones appear to be completely unmineralized, which would quickly be fatal due to respiratory failure.^120^ Infantile HPP patients always show a failure to thrive as well as rachitic features, including leg bowing, joint enlargement, rib fracture, progressive deformity of the thorax and tracheomalacia.^117,121,122^ Other signs such as hypercalcemia, muscle hypotonia, papilledema resulting from craniosynostosis and increased intracranial pressure can also be observed in some patients.^123,124^ Childhood HPP is the form with the greatest clinical variability. The infantile and childhood subtype are a continuum and are sometimes difficult to distinguish, with childhood HPP being more evident in most cases.^123,125^ Delayed walking, a waddling gait, chronic bone and joint pain, recurrent fractures, scoliosis, shell teeth, hypoplasia of the cementum and caries in the permanent dentition are characteristic manifestations and common in childhood HPP.^126,127^ Adult HPP is always associated with an early loss of permanent dentition and painful feet or hips due to femoral pseudofractures in the lateral cortices of the femora (Looser’s zones) and recurrent metatarsal stress fractures.^128–130^ Pyrophosphate arthropathy, pseudogout, spinal hyperostosis and calcific periarthritis are also observed in some adult HPP patients.^121,131,132^

There is no established medical management for HPP, but only therapeutic interventions that consist of symptom palliation, calcium balance maintenance and physical, occupational, dental and orthopaedic interventions, as necessary, to alleviate symptoms and reduce complications. Regarding tracheomalacia and pulmonary hypoplasia in infantile and childhood HPP, mechanical ventilation is necessary,^133^ and to avoid neurological complications, vitamin B6 or pyridoxine supplementation, and/or craniectomy if necessary, is recommended.^134,135^ Concerning pseudofractures or completed fractures in adult HPP, the use of teriparatide (recombinant human parathyroid hormone), load-sharing intramedullary fixations or ankle-foot orthoses is often applied, and naproxen might be used to diminish skeletal pain.^130,136,137^ It is worth noting that bisphosphonate treatment for ‘osteoporosis’ in HPP patients is ineffective, but PTH provides some mitigation.^138^ Additionally, dental prosthetic interventions (especially removable partial dentures in childhood) are well recommended to facilitate the normal development of speech and avoid abnormal transversal development of the jaw and related social problems.^117^ Currently, several newly proposed treatments in infants and young children, such as bone marrow and mesenchymal stem cell transplantation^139^ and enzyme replacement therapy (ERT, Asfotase alfa),^133,140^ have achieved promising results for the bones and lungs.

#### Marfan syndrome

Marfan syndrome (MFS) is a genetic autosomal dominant disorder of the connective tissue and is caused by mutations in the *FBN1* gene, which encodes extracellular matrix protein fibrillin-1.^141^ Several studies have demonstrated that the transforming growth factor β (TGFβ) signalling system may also be involved in the development of MFS.^142,143^ MFS can manifest either at birth or as a progressive disease that can be found as late as 30–40 years old.

Several signs and symptoms have been reported to be associated with MFS, especially the skeletal, cardiovascular and ocular systems.^144^ In the skeletal system, most individuals with MFS present arachnodactyly (positive Walker Murdoch sign and Steinberg sign), dolichostenomelia and deformity of the spine and chest wall, due to disproportionate linear overgrowth of tubular bones, and some MFS patients demonstrate osteopenia, ligament laxity, hindfoot valgus with forefoot abduction, a high risk of knee and ankle sprains, and protusio acetabuli. All these manifestations usually appear during childhood and may worsen during adolescence. Additionally, typical craniofacial deformities, such as a long narrow skull, high arched palate with crowing teeth, midface hypoplasia, mandibular retrognathia and malar hypoplasia, are also observed in MFS patients^145,146^ (Table 2). In the cardiovascular system, enlargement of the aortic root, pulmonary artery dilatation, valvulopathies, cardiomyopathy due to elastic fibre degeneration in the aorta, and subsequent abdominal aortic aneurysms and progressive myocardial dysfunction are common in MFS patients and are always associated with severe consequences for survival.^147–150^ In ocular and other systems, ectopia lentis, near-sightedness, intracranial hypotension-associated headache due to dural ectasia, spontaneous pneumothorax and striae atropicae on the arms, hip and lower back, are very frequent in MFS patients.^151–154^

Cardiac and pulmonary impactions are the most severe complications of MFS, while osteopenia and bone pain are the two most poorly managed features of MFS, particularly in elderly patients. Although there is no cure for MFS, life expectancy can be significantly increased after a life-style modification, such as reducing emotional stress and restricting physical activities^155^ and undergoing regular echocardiographic imaging assessments,^156^ pharmacological treatment (propranolol and other β-blockers, calcium channel blockers, angiotensin receptor blockers and angiotensin-converting enzyme inhibitors)^157,158^ and prophylactic surgery (replacement of the aortic valve and ascending aorta, vitreolensectomy with laser prophylaxis).^159^ For MFS patients with severe craniofacial deformities, combined orthognathic and orthodontic treatment is recommended to correct crowing teeth and mandibular retrognathia.

#### McCune–Albright syndrome

McCune–Albright syndrome (MAS) is a non-hereditary genetic disorder this is caused by a spontaneous postzygotic mutation of the gene *GNAS*, which is involved in G-protein signalling and results in constitutive activation of Gsα protein as well as the production of excess cAMP^160,161^ The *GNAS* mutation arises very early during embryogenesis and occurs only in the mosaic state, which leads to a variable pattern in affected tissues.^162^

MAS was initially defined as the triad of polyostotic fibrous dysplasia (FD) of the bone, café-au-lait skin pigmentation and precocious puberty,^163^ and over years it has been refined as a disorder involving at least one of the following clinical manifestations: café-au-lait macules, fibrous dysplasia, and autonomous endocrine hyperfunction.^163^ The light brown café au lait macules arising from the ectoderm are often the first observed feature of MAS patients, which usually appear at or shortly after birth. They are described as having an irregular border and mostly affect the midline of the body, such as the posterior neck, base of the spine and face.^164^ Additionally, oral mucosal pigmentation has been documented in a minority of MAS patients.^165^ FD arises from the mesoderm and can occur in one bone as well as a combination of craniofacial-axial bones and the appendicular skeleton as a hallmark of MAS. The affected bones are characterized by extensive lesions with a thin cortex and ‘ground glass’-like intramedullary matrix.^166^ Craniofacial deformities may present as a facial asymmetry due to an expanding FD lesion, which may progress along with dental malocclusion, hearing impairment and vision changes.^167,168^ Craniofacial FD is also associated with tooth development and dental disorders, such as dentin dysplasia, taurodontism and a high caries index, and therefore, more frequent scaling and root planing as well as topical fluoride application are required to control dental plaque and caries in MAS patients.^169,170^ Jaw FD may contribute to aneurysmal bone cysts and osteosarcoma^171,172^ (Table 2). FD-involved sphenoid bone may lead to a pituitary adenoma, and conversely, excess growth hormone can worsen craniofacial bone disease.^173,174^ Limb deformities usually present both valgus and varus deformities in both knees, appendicular skeletal fractures as well as curved femurs.^162,175^ Additionally, scoliosis appears to occur frequently in MAS patients.^176^ Endocrine hyperfunction in MAS patients consists of several disorders, including precocious puberty, Cushing’s syndrome, excess growth hormone and prolactin, renal phosphate wasting, and hyperthyroidism, and it is more common in females than males. Thus, growth acceleration, testicular enlargement or Sertoli cell tumours, vaginal bleeding or spotting, hypophosphatemia-related rickets or fractures, advanced skeletal maturity and acromegaly may occur in most MAS patients.^164,168^ Additionally, hepatic and cardiac complications have also been reported in MAS patients.^163^

The affected multi-organ clinical pattern makes the management of MAS complex and challenging. Regarding FD, physical therapy is used to maintain the strength and range of motion of affected bones,^177^ surgical treatment involves orthopaedic surgery or orthognathic surgery combined with orthodontic therapy, lesion resection surgery and intramedullary device fixation, which are applied for FD associated craniofacial and limb deformities as well as fractures,^170,178^ and antiresorptive therapy using bisphosphonates is also advocated to relieve FD-related bone pain and reduce bone resorption.^179,180^ However, surgical management is not recommended in most patients, and there is no satisfactory treatment capable of altering the progress of FD in MAS patients. Thus, annual observation may be necessary. Concerning endocrinopathies, medical treatment is mainly used. Somatostatin analogues such as pegvisomant are used to treat excess growth hormone, the aromatase inhibitor letrozole combined with leuprolide or testosterone receptor antagonist is used for precocious puberty, and oral phosphorus and calcitriol supplementation is used for hyperthyroidism.^177,181^ In addition to medical treatments, several surgeries, including thyroidectomy and hypophysectomy, have been reported as a potential option.^168,182^ Treatment for skin hyperpigmentation in MAS patients is not routinely conducted, although laser therapy can be applied with satisfactory results.^182^

#### Kallmann syndrome

Kallmann syndrome (KS) is a form of a group of genetic disorders termed isolated gonadotropin-releasing hormone (GnRH) deficiency (IGD).^183^ Hypogonadotropic hypogonadism (HH) occurs with anosmia and can be termed KS.^184^ Although the genetic defect in most IGD patients remains uncharacterized and involves a sequence variant of several different genes, the genes that regulate neurodevelopmental IGD pathways, including neural cell adhesion and axonal migration, have been reported to contribute to KS. Such genes include Kallmann 1, 2 (*KAL1, 2*), NMDA receptor synaptonuclear signalling and neuronal migration factor (*NSMF*), fibroblast growth factor receptors 1, 8 and 17 (*FGF1, 8, 17*), semaphorin 3A (*SEMA3A*), SRY Box 10 (*SOX10*) and many more.^185^

The clinical features of KS can be divided into two parts: reproductive features and non-reproductive features. Among the reproductive features, KS patients are characterized by low blood levels of sex hormones, gonadotropins and subsequent failure of puberty onset, low libido and poor sexual function, as well as infertility. Other signs such as a micropenis and lack of testicular development or cryptorchidism in males and delayed menarche and failure to start menstruation in females have also been observed in some KS patients.^184,186^ Regarding non-reproductive features, a wide spectrum of clinical manifestations can be involved, most of which are associated with mutations in different genes. In addition to a partial or total lack of sense of smell due to olfactory placode cell migration from the nasal region to inside the hypothalamus, cleft palate, hare lip, a high-arched palate and other midline craniofacial defects, hypodontia, malformed teeth and other dental abnormalities, short metacarpals, scoliosis, neural hearing loss, bimanual synkinesis due to cerebellar ataxia, eye movement disorders, colour blindness, unilateral renal agenesis and other maladies are also frequently observed in KS patients^186–192^ (Table 2). In addition, as a result of a deficiency in either testosterone or oestrogen, which is important to maintain bone density, KS patients are exposed to a higher risk of developing secondary osteoporosis or osteopenia.^193,194^ Thus, an increased tendency toward fracture may be observed in these patients.

The treatment for KS patients aims first to initiate the development of secondary sexual characteristics and second, to develop fertility, sex hormone replacement in childhood and gonadotropin and GnRH pulsatile therapy in adolescence and adulthood are the most frequently applied treatments to stimulate virilization or oestrogenization and restore fertility.^195^ Early surgical corrections before 1 year of age are recommended for KS patients with cryptorchidism.^196^ Other skeletal phenotypes such as a cleft palate and lip and hypodontia require related repair surgery, and patients with tooth agenesis or hypodontia require dental restoration early in life.^187^

#### Fanconi anaemia

Fanconi anaemia (FA) is a genetic autosomal recessive genetic disorder that results from several FA or FA-like genes, such as *FANCA, -C, -D, -E, -F, -G, -J, -L*, *-M, -N, -P, -S* and *XPF*, all which are involved in an impaired response to DNA damage.^197–200^ The proteins encoded by these genes and their complex have been reported to protect cells from oxidation-induced genotoxicity and to directly participate in STAT pathway activation and protein kinase pathway suppression, which is important in regulating the apoptosis of hematopoietic cells.^200–204^ Consequently, haematologic components, including blood cells and platelets, fail to develop, and bone marrow failure syndromes gradually develop.

Patients with FA are characterized by bone marrow failure, myelodysplastic syndromes/acute myeloid leukaemia (MDSs/AML), typical skeletal deformities and an increased incidence of solid tumours. Skin discolorations such as petechiae, bruises and cafe´-au-lait spots are usually the first signs of haematologic problem in FA patients.^205^ Subsequently, FA patients may exhibit a pale appearance, feel tired, and develop infections, 20% of whom may develop MDSs/AML during their teens or young adulthood due to hypoplastic or aplastic anaemia or cytopenia, unexplained macrocytosis and bone marrow failure.^205,206^ Furthermore, facial and skeletal abnormalities are frequently observed in patients with FA, as well as short stature, fanconi facies (microcephaly, microphthalmia and ptosis, microphthalmia and triangular face), and abnormal radii and thumbs (clinodactyly and polydactyly)^199,207–210^ (Table 2). Other symptoms such as the onset of solid tumours or cancers (especially head and neck squamous cell carcinomas, HNSCCs), abnormal reproductive organs with reduced fertility, renal and urinary tract problems, heart defects and gastrointestinal problems have also been reported, but with a relatively lower incidence.^199,211^

Currently, the most common management for FA involves HSCT and the application of androgens and hematopoietic growth factors. HSCT is the only way to establish normal haematopoiesis and can significantly extend patients’ lifespan; however, not all patients are candidates for transplantation, and the high solid tumour incidence has not been addressed.^199,212–214^ Due to the severe cytotoxicity of chemoradiotherapy for FA patients, surgical resection is preferred, while HPV vaccination and frequent dental evaluations or bone marrow aspirates are recommended to rule out early cancers.^199,211^ Additionally, induced pluripotent stem cell transplantation and gene therapy for FA have recently been proposed with promising pre-clinical results.^215,216^

### Skin, mucosa and soft tissue abnormalities

#### Hereditary epidermolysis bullosa

Hereditary epidermolysis bullosa (EB) comprises a group of rare genetic disease characterized by increasing skin fragility, which results in blisters or erosions of the skin and mucous membranes in response to minor mechanical injury, such as scratching.^217^ Mutations or errors in the genetic code lead to a defect in attachment between or within two layers of the skin-epidermis and dermis, thus resulting in extremely fragile skin.^218^ Currently, mutations in at least one of twenty different genes has been found to cause a large spectrum of phenotypes in EB patients, ranging from mild to lethal.^219^ Based on EB-related genes for proteins with different cellular localizations (intracellular, transmembrane or extracellular), EB is categorized into four major types: epidermolysis bullosa simplex, junctional epidermolysis bullosa, dystrophic epidermolysis bullosa, and Kindler syndrome.^218,220^ Although the types differ in signs and symptoms, the underlying mechanism is identical. These mutations of specific genes prevent the production of essential proteins to strengthen the skin or produce antibodies against structural components of the skin. Basal variants of epidermolysis bullosa simplex (EBS), the most common EB type, are frequently caused by dominant negative missense mutations in the KRT5 or KRT14 genes.^221^ These genes supply instructions for producing tough, fibrous proteins to provide resiliency to the epidermis. Mutations in either the KRT5 or KRT14 gene will lead to destabilization of the cytoskeleton and cytolysis upon mechanical stress. Similarly, junctional epidermolysis bullosa (JEB) results from mutations in the LAMA3, LAMB3, LAMC2, and COL17A1 genes, which lead to defects in the production of laminin 332, an important protein to help attach the epidermis to the dermis. Dystrophic epidermolysis bullosa (DEB) is caused by mutations in the gene encoding collagen VII, the main structural component anchoring the epidermis to the dermis. Defective anchoring fibrils will eventually result in separation of the sub-basal lamina.^222^ Kindler syndrome derives from mutations in the FERMT1 gene. This gene provides instructions for producing a protein called kindlin-1. A lack of this protein leads to the disruption of many essential cell functions. For example, the structure of keratinocytes can be abnormal without kindlin-1, and proliferation and cell division can be disrupted simultaneously. Such changes would cause the skin to be fragile and prone to blistering.^223^

EB may lead to marked oral involvement, yet the extent and features vary greatly from one EB type to another. In the mild forms, the oral mucosa may only suffer discrete blistering that heals rapidly without scarring. However, in more severe cases, the entire oral mucosa can be affected and severe intraoral blistering observed with subsequent scar formation. Extensive intraoral scarring will even cause ankyloglossia and obliteration of the oral vestibule.^224^ In different EB types, the dentition may also be severely affected by enamel hypoplasia and/or caries. The prevalence of enamel hypoplasia has been found to be higher in patients with JEB than other EB types,^225^ yet all EB patients are prone to enamel hypoplasia compared with normal controls.^226^ Rampant dental caries in patients with JEB seems to occur due to enamel hypoplasia, which decreases the intrinsic resistance of the tooth. However, despite normal dentition development, rampant dental caries are also frequently detected in DEB patients, which could be attributed to the special diet (soft) and limited oral clearance (limited tongue and cheek mobility) of EB patients (Table 3). Apart from easily blistering skin and mucous membranes, complications of epidermolysis bullosa may include the following: anaemia, muscular dystrophy, dysphagia, pyloric atresia, constipation, cardiomyopathy, renal insufficiency, syndactyly, osteoporosis and a high risk of cancer.^220,225^

**Table 3:** Dental-craniofacial manifestations of skin, mucosa and soft tissue abnormality-related rare diseases

Rare diseases	Aetiology	Major manifestations	Dental-cranio-facial manifestations	Incidence^a^	Onset period^b^
Hereditary epidermolysis bullosa	Defect in attachment between the epidermis and dermis of the skin	Hands and feet blisters at the site of rubbing	Intraoral blistering with or without scar formation, oral vestibule; Enamel hypoplasia and/or caries	38.6%–94.8% 18.1–100%	Perinatal period Early childhood
Peutz–Jeghers syndrome	Mutations in the *LKB1* gene	Benign hamartomatous polyps in the gastrointestinal tract; skin hyperpigmented macules in hand and feet	Hyperpigmented macules in lip and oral mucosa (Gingiva, hard palate and inside of the cheek).	90%–95%	Infanthood
Mucopolysaccharidosis	Absence or malfunctioning of lysosomal enzymes	Developmental delay, intellectual disabilities, short stature; impaired motor function; hearing loss; respiratory distress, obstructive sleep apnoea; enlarged or diseased heart valves	High-arched palate, hypertrophy of the alveolar processes; Enlarged tongue, gingiva and associated anterior open bite; Delayed tooth eruption, impacted teeth	56.3%–85.7% 70%–86.7% 75%–85.7%	Childhood Childhood Early childhood
Mikulicz's disease	Abnormal IgG4 deposition and related inflammation	Continuous painless lacrimal gland swelling; pulmonary interstitial fibrosis	Painless and persistent parotid, submandibular and sublingual salivary glands swelling	54.5%–100%	Adulthood
Primary light-chain amyloidosis	Abnormal light chains deposition	Renal failure; heart failure; enlarged liver	Macroglossia, submandibular swelling	8%–20%	Adulthood

^a^Incidence of dental-craniofacial manifestations

^b^Onset period of dental-craniofacial manifestations

EB has no effective therapy or cure at present. Although the longevity of patients with mild forms may not be affected significantly by EB, multiple interventions from a range of medical specialists are required to ensure quality of life. The current treatment largely targets symptoms and focuses on caring for and preventing the formation of new blisters. The daily management available for EB patients includes wound care, pain management, and protective bandaging. Attention must be paid to improve nutrition and help with weight gain.^217,222,226^ For patients with severe EB, surgical treatment, such as widening of the oesophagus, placement of a feeding tube and skin grafting, is optional.^227,228^ Additionally, working with a rehabilitation specialist may help relieve the limitations on motion induced by scarring. Moreover, when performing therapies, such as restorative dental treatment, local anaesthesia, mucosal lubrication with hydrocortisone cream or triamcinolone are recommended to relieve severe oral and perioral scarring, microstomia, ankyloglossia or limited mouth opening. Furthermore, instructions on toothbrushing and dietary habits and a comprehensive assessment of caries risk or activity are also strongly suggested to reduce the likelihood or severity of caries disease.^224,226^

#### Peutz–Jeghers syndrome

Peutz–Jeghers syndrome (PJS) is an autosomal dominant genetic disease that is mainly caused by mutations (deletion, insertion, or single base pair substitutions) in the *LKB1* gene on chromosome 19p13.3, which encodes serine-threonine kinase 11 (STK11) and may function as a tumour suppressor.^229^ STK11 has been reported to regulate cellular proliferation, apoptosis and to play an important role in cell polarity, cell metabolism and energy homoeostasis.^230–232^ The function of *LKB1* is complex and is still being investigated, and no clear genotype-phenotype correlation has been identified in PJS patients.^233^

PJS is characterized by intestinal benign hamartomatous polyps combined with intermittent abdominal pain, as well as hyperpigmented macules that vary from 1 to 5 mm in size on the skin (nose, periorbital, back of hands, and tips of toes and fingers), lip and oral mucosa (gingiva, hard palate and inside the cheek are most frequently involved).^234^ Mostly mucocutaneous pigmentation appears early in childhood, before the onset of gastrointestinal disease, and gradually disappears after puberty, but oral lesions persist throughout life and are usually flat and painless.^235^ The size and colour of the pigmentations are not affected by sunlight, unlike regular ephelides (Table 3). Moreover, although hamartomatous polyps have an extremely low potential for malignancy, patients with PJS have an almost 15-fold higher tendency to develop cancer in parts of the body, such as the pancreas, liver, lungs, breast, ovaries, uterus, testes and other organs.^236,237^ Additionally, bowel obstruction, intussusception and iron-deficiency anaemia due to profound gastrointestinal bleeding are also frequently reported in PJS patients.^238,239^

The management of PJS consists of the surveillance and treatment of the hamartomatous polyps. Resection of the polyps is performed only when serious bleeding occurs, and enterotomy is usually performed to resect large nodules.^235^ As no standard treatment is currently established for mucocutaneous pigmentation, cryosurgery, electrodessication and the Q-switched ruby laser have been used to remove these lesions but consistently result in unsuccessful removal and scarring.^240^ Surveillance in PJS patients aims to detect sizable gastroenterological polyps as well as cancer at an early stage to reduce the likelihood of potential polyp-related complications, such as intussusception, and improve the survival and cure rate of potential cancer. Therefore, annual clinical examination and testicular examination from birth until 12 years of age, colonoscopy and video capsule endoscopy beginning at 8 years of age, and annual breast MRI beginning at 25 years of age, among others, are recommended.^233^

#### Mucopolysaccharidosis

Mucopolysaccharidoses (MPS) are a group of genetic metabolic disorders resulting from the absence or malfunction of acid hydrolase, a kind of lysosomal enzyme that is required to break down sulphated glycosaminoglycans (GAGs).^241^ GAGs are series of long chains of sugar carbohydrates that are present on both cell surfaces and in the extracellular matrix in all tissues, such as heparan sulphate, keratan sulphate, chondroitin sulphate and many more. Over time, due to the deficiency of lysosomal enzymes, GAGs accumulate in the cells, blood and connective tissues and subsequently result in cellular damage and tissue and organ dysfunction.^242^ There are currently 11 known enzyme deficiencies resulting from more than 40 genetic disorders that account for 7 distinct MPS types.^243^

Different MPS types share several clinical phenotypes but have various degrees of severity, and not all of the features may be apparent at birth but may progress as GAGs start to accumulate.^242^ Each individual disorder has a wide spectrum of clinical features, including skeletal dysostosis (prominent forehead, dwarfism with a short neck and short trunk, claw hands, joint stiffness and spinal dysostosis), neurologic manifestations (hearing loss, speech and language delay, intellectual disabilities and learning deficiency, declining neurological status), motor dysfunction (hyperactivity, behavioural issues and motor impairment) and other somatic symptoms (respiratory distress, hepatomegaly, hernias and excessive body hair growth).^243–251^ Among the different types of MPS, type II shows significant CNS involvement, type III shows severe motor dysfunction, and type IV and VI shows more prominent skeletal disorders, while the others present both.^242^ Additionally, distinct facial manifestations have also been reported in most MPS patients, especially in type I and VI, such as rough skin, bulging forehead, depressed nasal bridge, enlarged mouth and thick lips.^252^ In the oral cavity, GAG accumulation would contribute to an enlarged tongue, gingiva and associated anterior open bite, high arched palate, hypertrophy of the alveolar processes, delayed tooth eruption and the formation of dental follicles^253–255^ (Table 3).

The current treatments include allogeneic HSCT and enzyme replacement therapy, which is only available for MPS type I, III and VI. ERT can easily reach the reticuloendothelial organs and significantly reduce GAGs and related inflammatory cytokines, but it provides limited improvement of joint pain stiffness and other skeletal changes.^256,257^ Thus, ERT is considered to be effective in reducing the development of pathology but not reversing established deformities. HSCT is only recommended for young children with severe MPS, as the application of HSCT can promote the establishment of intellectual development and functions but shows limited efficacy in preventing osteoarticular deformities.^258–261^ Additionally, other therapies, including the application of active site-specific chaperones that increase residual enzyme activity or genistein that inhibits GAG synthesis (substrate reduction therapy), nonsteroidal drug administration for anti-inflammatory treatment, surgery interventions for correcting deformities or respiratory status, and gene therapy referring to inserting the wild copy of the defective genes, have recently been proposed and shown promising results in clinical or pre-clinical trials.^262–266^ For patients with severe occlusal characteristics, such as a marked overjet, anterior open bite and mandibular protrusion, orthodontic with or without orthognathic, surgery should be considered to improve quality of life after ERT.^255^

#### Mikulicz's disease

Mikulicz’s disease (MD) was first described in 1892 by Mikulicz as a developmental swelling disorder of the lacrimal and salivary glands.^267^ In recent past, MD has been considered a part of Sjogren syndrome, but with the development of specific laboratory examinations, it is now accepted as part of IgG4-related disease (IgG4-RD) and distinct from Sjogren syndrome.^268^ IgG4-RD is a chronic inflammatory condition that is characterized by Th2 and regulatory immune reactions, manifesting as high serum levels of IgG4 and tissue infiltration with lymphocytes and IgG4-secreting plasma cells.^269,270^ However, the physiological role of IgG4 in IgG4-RD remains unclear.

Clinically, MD patients present dry eye, exophthalmos and dry mouth, as well as continuous and painless bilateral and symmetrical enlargement of the related glands, while most MD patients do not have keratoconjunctivitis sicca.^271,272^ MD usually involves the parotid, submandibular and occasionally sublingual salivary glands, but minor salivary gland involvement can also be observed. Nelson reported a MD patient with only nonulcerated, painless, irregular swelling of the left hard palate in 1963^273^ (Table 3). Additionally, MD occasionally appears in combination with other IgG4-RD, including autoimmune pancreatitis, Riedel’s thyroiditis, Küttner’s tumour and other extra salivary and lacrimal gland lesions.^274–277^

Currently, MD is mainly treated with immunosuppressive therapy, and it shows a good response to the administration of steroids, including a rapid improvement in glandular swelling and salivary secretion.^278,279^ However, relapse frequently occurs in the absence of therapy.^279^

#### Primary light-chain amyloidosis

Primary light-chain amyloidosis (AL) is caused by the developmental amyloid deposition of abnormal protein fibres (misfolded free immunoglobulin or λ light chains), which can lead to structural and functional damage of different organs, such as the heart, kidneys, liver and brain.^280^ Skin and mucous membrane changes, including purpura, petechiae, ecchymoses and bullous lesions, are often the first features of primary AL patients, while weight loss and severe fatigue usually occur in most primary AL patients.^281–283^ Additionally, other symptoms are also frequently reported, such as heart failure, renal failure or end-stage renal disease, hepatomegaly without scan defects and postural hypotension, and cardiac involvement is the main determinant of survival.^284,285^ Macroglossia, submandibular swelling, alopecia and shoulder pain are not common in primary AL patients but are usually associated with very advanced disease^286^ (Table 3). The treatment of primary AL must be adapted to the heterogeneous manifestations, and combination chemotherapy, such as melphalan and dexamethasone or bortezomib and dexamethasone, as well as cyclophosphamide, have been reported to be applied in patients who are ineligible for autologous bone marrow transplants.^287–289^ Additionally, in severe patients with heart or rental failure, renal and cardiac transplantation may improve quality of life and prolong the lifespan.^288^

### Others

#### Angelman syndrome

Angelman syndrome (AS) is a genetic disorder resulting from a new maternal mutation, deletion or imprinting defect in chromosome 15q11-q13, which contains the *UBE3A* and *OCA2* gene, rather than being inherited from the parents.^290^ Because the paternal inherited allele of *UBE3A* is epigenetically silenced in most neurons, maternal deletions of *UBE3A* result in a nearly total selective loss of brain *UBE3A* function.^291^ The *UBE3A* gene encodes a HECT (homologous to E6-associated protein C terminus) domain E3 ubiquitin ligase, which ubiquitinates protein substrates such as p53, p27, Pbl/Ect2, Ephexin5 and many more, and leading to their degradation. Multiple mutations in UBE3A contribute to a defective catalytic function and result in disorders when regulating neuronal apoptosis, differentiation and axon outgrowth.^291^

AS is characterized by childhood epilepsy and severe developmental delay with or without mental retardation. Developmental delay in AS individuals is usually observed before the first year of life, which manifests as decreased sleep; speech impairment; poor oral-motor functions, such as sucking and chewing; movement or balance disorders, such as tremors and ataxia; and behavioural uniqueness, such as a happy demeanour, excitability or hyperactivity.^292,293^ Seizures usually start between 1 and 3 years of age, which first only present as mild myoclonic jerks and spells of atypical absences and then become more frequent in early child hood, tending to decrease in adolescence.^294^ A typical pattern of large-amplitude slow-spike waves can be noted and used for diagnosis using an electroencephalogram.^295^ Additionally, a tendency to develop flexion contractures and valgus deformity of the feet, scoliosis and imperfect manual function are also observed with age in some individuals with AS.^296^ Several dental-craniofacial deformities in AS patients have also been reported in several studies, although with a reduced incidence, including a flat occiput, protruding tongue, mandible prognathia, wide mouth and wide-spaced teeth^295^ (Table 4).

**Table 4:** Dental-craniofacial manifestations of other related rare diseases

Rare diseases	Aetiology	Major manifestations	Dental-cranio-facial manifestations	Incidence^a^	Onset period^b^
Angelman syndrome	Genetic mutations in chromosome 15q11-q13	Developmental delay; movement and balance disorder; behavioural uniqueness (excitability or hyperactivity); seizures; abnormal EEG	Unique behaviour: happy demeanour, poor oral function; Mandible prognathia, protruding tongue, wide mouth and wide-spaced teeth	75% —	Early childhood Childhood
Langerhans-cell histiocytosis	Abnormal proliferation of Langerhans type cells	lytic bone lesions; fever; diabetes insipidus (Hand-Schüller-Christian triad); scaly skin lesions in scalp, ear canals, and abdomen (Letterer–Siwe disease)	Ulcers, scabby and granuloma with pain and swelling at oral mucosae; Bone defect in craniofacial bones with punched-out appearance; Gingival necrosis with the movement of teeth	24% 55%–80% —	Childhood Childhood or adulthood —

EEG, electroencephalogram

^a^Incidence of dental-craniofacial manifestations

^b^Onset period of dental-craniofacial manifestations

Currently, there is no specific treatment for AS, and didactive-supportive therapy is generally applied with the aim of promoting mental development, such as educational training, speech training (the use of communication devices, adapted pictogram and modified sign language), and antiepileptic drug therapy, including sodium valproate and benzodiazepines for idiopathic generalized epilepsies.^292,293,295^ The remainder of treatment is applied according to the specific manifestations in individuals with AS: disordered sleep is treated with behavioural interventions and melatonin administration; behavioural modification is recommended for hypermotoric or hyperactive behaviours rather than drug therapy; special adaptive chairs or positioners and physiotherapy are provided for ataxic children; orthotic bracing or surgery for subluxed or pronated ankles, thoraco-lumbar jackets for severe scoliosia, and related orthodontic treatment, orthognathic surgery as well as dental restoration for correction of open bite, prominent diastema between the central incisors and other dental-craniofacial deformities are also supplied.^295^ Additionally, education about oral hygiene, such as regarding brushing techniques, should also be emphasized to maintain a caries-free state in the affected child.

#### Langerhans cell histiocytosis

Langerhans cell histiocytosis (LCH) is a developmental histiocytosis syndrome characterized by an abnormal proliferation of histiocytes. LCH is marked by excessive proliferation of Langerhans-type cells, which have immunophenotypic and ultrastructural similarities (CD1a antigen) to antigen-presenting Langerhans cells.^297^ The cause and pathogenesis of LCH remains a matter of debate concerning whether it is a kind of reactive or neoplastic process since Alfred Hand first described and misdiagnosed it as tuberculosis in 1983.^298^ The cytokine storm in the lesion region and high rate of spontaneous remissions support that LCH is an exaggerated physiological response of Langerhans cells,^299,300^ while the monoclonal proliferation of pathologic cells and a newly discovered potential somatic mutation of an oncogene, the BRAF gene, in LCH patients provides solid evidence that LCH is a malignancy.^301–303^

LCH consists of a wide spectrum of clinical disorders that vary from isolated bone lesions to multiple skeletal or visceral lesions, with or without lymph node involvement. Several clinical investigations have demonstrated that the peak incidence of LCH occurs between 1 and 3 years of age and that multiple-organ systematic diseases mostly begins before 2 years of age.^304^ At present, LCH is usually divided into two types according to the lesion scope: localized LCH (also called eosinophilic granuloma) and disseminated LCH (including Letter-Siwe disease and Hand–Schüller–Christian disease). Localized LCH consists of a simplex rash without affecting organs, simplex bone damage and multiple bone damage with or without diabetes insipidus.^305^ Diabetes insipidus is one of the most common manifestations in LCH patients due to the involvement of the central nervous system, including the hypothalamus and pituitary.^306^ Regarding rashes, invasive nodules and plaques or generalized seborrhoeic dermatitis-like rashes of the scalp, skin folds and retroauricular area are commonly reported. Additionally, LCH may also manifest as ulcers, scabs and granuloma with pain and swelling of the oral and genital mucosal region.^307^ Bone damage mainly involves the axial skeleton, such as the skull bones (orbit and temporal bone), mandible, pelvis and spine. Bone lesions of LCH demonstrate a combination of bone destruction and adjacent soft tissue mass, which can be easily observed using CT and MRI.^308^ The bone defect in skull bones usually displays a typical punched-out appearance with a scalloped or irregular margin and varies from poorly to well-defined. The superolateral and frontal part of the orbit and the tympanic, mastoid, and squamosal portions of the temporal bone are most frequently affected location in the skull bones.^309,310^ Multiple bone defects in the mandible can occur and initially present as a cystogranuloma around the teeth. The most common oral finding can be gingival necrosis with movement of the teeth and alveolar bone destruction.^311^ (Table 4) In patients with disseminated LCH, the internal organs are involved with or without dysfunction of the lung, liver or hematopoietic system.^312^ Several systematic symptoms such as lymphadenopathy, hepatosplenomegaly and anaemia can be observed in the early stage of disseminated LCH patients.

Treatments for LCH are variable depending on the localization and number of lesions, and the aim of treatment is to correct the organ dysfunction as well as to limit the spread of disease. For localized LCH, surgical resection and curettage with or without bone grafting combined with topical steroids or vinblastine are the first-line therapy.^313^ For disseminated LCH, systemic therapy consisting of steroids and vinblastine is recommended^314,315^; cytarabine or HSCT are used as second-line therapy in patients who do not response to the steroids and vinblastine.^316,317^ Furthermore, chemotherapy and radiotherapy can also be used alone or in combination for disseminated LCH, but this treatment paradigm is controversial, and strong chemoradiotherapy is not recommended to avoid severe toxic and side effects.^311^ In addition, a BRAF inhibitor, vemurafenib, was recently approved by the US Food and Drug Administration to treat severe BRAF mutation-positive LCH patients and showed substantial clinical and biological improvements without severe toxicities or adverse events.^318^

## Conclusion

Approximately 7 000 rare diseases have been identified around the world, which far exceeds the recently defined 121 rare diseases in China. However, not all rare diseases have effective diagnosis or treatment regimens, and in consideration of the limited resources, it is much more meaningful to focus on the rare diseases with relatively a higher incidence as well as feasible management or precaution tactics. Based on the established ‘Rare Diseases List in China’, it is important for our clinicians to fully comprehend the related clinical features and potential treatments for early diagnosis and management as well as for improving the patient’s prognosis. Moreover, several rare diseases show unique dental-craniofacial manifestations in the early disease period, such as congenital erythropoietic porphyria, hypophosphatasia, Marfan syndrome, and Peutz–Jeghers syndrome, among others (Tables 1–4); thus, dentists and oral maxillofacial surgeons could be the first clinicians to identify these rare diseases at an early stage, making it particularly necessary for them to adequately grasp the related clinical features.

## References

[1] Dawkins HJS (2018). Progress in rare diseases research 2010-2016: an IRDiRC perspective. Clin. Transl. Sci..

[2] Aymé S, Schmidtke J (2007). Networking for rare diseases: a necessity for Europe. Bundesgesundheitsblatt. Gesundh. Gesundh..

[3] Azie N, Vincent J (2012). Rare diseases: the bane of modern society and the quest for cures. Clin. Pharmacol. Ther..

[4] Schieppati A, Henter JI, Daina E, Aperia A (2008). Why rare diseases are an important medical and social issue. Lancet.

[5] Melnikova I (2012). Rare diseases and orphan drugs. Nat. Rev. Drug. Discov..

[6] Ekins S (2017). Industrializing rare disease therapy discovery and development. Nat. Biotechnol..

[7] James, W. D., Elston, D. M. & Berger, T. G. *Andrews' Diseases of Skin: Clinical Dermatology*, 12e. (Philadelphia: Elsevier, 2016).

[8] Pinheiro M, Freire-Maia N (1994). Ectodermal dysplasias: a clinical classification and a causal review. Am. J. Med. Genet. A..

[9] Halai T, Stevens C (2015). Ectodermal dysplasia: a clinical overview for the dental practitioner. Dent. Update.

[10] Lexner MO, Bardow A, Hertz JM, Nielsen LA, Kreiborg S (2007). Anomalies of tooth formation in hypohidrotic ectodermal dysplasia. Int. J. Paediatr. Dent..

[11] Leao JC, Ferreira AMC, Bandeira V, Figueirôa FV, Porter SR (2005). Anhydrotic ectodermal dysplasia (Christ-Siemens-Touraine syndrome). A case report. Int. Dent. J..

[12] Crawford PJ, Aldred MJ, Clarke A (1991). Clinical and radiographic dental findings in X linked hypohidrotic ectodermal dysplasia. J. Med. Genet..

[13] Tso MS, Crawford PJ, Miller J (1985). Hypodontia, ectodermal dysplasia and sweat pore count. Br. Dent. J..

[14] Nakata M, Koshiba H, Eto K, Nance WE (1980). A genetic study of anodontia in X-linked hypohidrotic ectodermal dysplasia. Am. J. Hum. Genet..

[15] Borzabadi-Farahani A (2012). Orthodontic considerations in restorative management of hypodontia patients with endosseous implants. J. Oral. Implantol..

[16] Rad AS, Siadat H, Monzavi A, Mangoli AA (2007). Full mouth rehabilitation of a hypohidrotic ectodermal dysplasia patient with dental implants: a clinical report. J. Prosthodont..

[17] Bergendal B (2010). Oligodontia ectodermal dysplasia—on signs, symptoms, genetics, and outcomes of dental treatment. Swed. Dent. J. Suppl..

[18] Lind LK, Stecksén-Blicks C, Lejon K, Schmitt-Egenolf M (2006). EDAR mutation in autosomal dominant hypohidrotic ectodermal dysplasia in two Swedish families. Bmc. Med. Genet..

[19] Singh P, Warnakulasuriya S (2004). Aplasia of submandibular salivary glands associated with ectodermal dysplasia. J. Oral. Pathol. Med..

[20] Nordgarden H, Storhaug K, Lyngstadaas SP, Jensen JL (2003). Salivary gland function in persons with ectodermal dysplasias. Eur. J. Oral. Sci..

[21] Pober BR (2010). Williams–Beuren syndrome. N. Engl. J. Med..

[22] Stasia MJ (2013). Functional and genetic characterization of two extremely rare cases of Williams–Beuren Syndrome associated with chronic granulomatous disease. Eur. J. Hum. Genet..

[23] Zhukova N, Naqvi A (2013). Williams-Beuren syndrome and burkitt leukemia. J. Pediatr. Hematol. Oncol..

[24] Vandeweyer G, Van der Aa N, Reyniers E, Kooy RF (2012). The contribution of CLIP2 haploinsufficiency to the clinical manifestations of the Williams-Beuren syndrome. Am. J. Hum. Genet..

[25] Crespi BJ, Hurd PL (2014). Cognitive-behavioral phenotypes of Williams syndrome are associated with genetic variation in the GTF2I gene, in a healthy population. Bmc. Neurosci..

[26] Martens MA, Wilson SJ, Reutens DC (2008). Research review: Williams syndrome: a critical review of the cognitive, behavioral, and neuroanatomical phenotype. J. Child Psychol. Psychiatr..

[27] Kapp ME, von Noorden GK, Jenkins R (1995). Strabismus in Williams syndrome. Am. J. Ophthalmol..

[28] Olsen RK (2009). Retinotopically defined primary visual cortex in Williams syndrome. Brain.

[29] Van der Geest JN (2005). Visual depth processing in Williams–Beuren syndrome. Exp. Brain Res..

[30] Morris CA (2010). Introduction: Williams syndrome. Am. J. Med. Genet. C. Semin. Med. Genet..

[31] Kruszka P (2018). Williams-Beuren syndrome in diverse populations. Am. J. Med. Genet. A..

[32] Torres CP (2015). Oral findings and dental treatment in a child with Williams-Beuren syndrome. Braz. Dent. J..

[33] Meyer-Lindenberg A, Mervis CB, Berman KF (2006). Neural mechanisms in Williams syndrome: a unique window to genetic influences on cognition and behaviour. Nat. Rev. Neurosci..

[34] Carrasco X, Castillo S, Aravena T, Rothhammer P, Aboitiz F (2005). Williams syndrome: pediatric, neurologic, and cognitive development. Pediatr. Neurol..

[35] Haas BW (2009). Genetic influences on sociability: heightened amygdala reactivity and event-related responses to positive social stimuli in Williams syndrome. J. Neurosci..

[36] Dykens E (2003). Anxiety, fears, and phobias in persons with Williams syndrome. Dev. Neuropsychol..

[37] Fahim C (2012). Williams syndrome: a relationship between genetics, brain morphology and behaviour. J. Intellect. Disabil. Res..

[38] Martens MA, Reutens DC, Wilson SJ (2010). Auditory cortical volumes and musical ability in Williams syndrome. Neuropsychologia.

[39] Wengenroth M, Blatow M, Bendszus M, Schneider P (2010). Leftward lateralization of auditory cortex underlies holistic sound perception in Williams syndrome. PLoS One.

[40] Järvinen A, Korenberg JR, Bellugi U (2013). The social phenotype of Williams syndrome. Curr. Opin. Neurobiol..

[41] Knowler WC, Pettitt DJ, Saad MF, Bennett PH (1990). Diabetes mellitus in the pima indians: incidence, risk factors and pathogenesis. Diabetes Metab. Rev..

[42] Partsch CJ (1999). Longitudinal evaluation of growth, puberty, and bone maturation in children with Williams syndrome. J. Pediatr..

[43] Palacios-Verdú MG (2015). Metabolic abnormalities in Williams–Beuren syndrome. J. Med. Genet..

[44] Cherniske EM (2004). Multisystem study of 20 older adults with Williams syndrome. Am. J. Med. Genet. A..

[45] Geggel RL, Gauvreau K, Lock JE (2001). Balloon dilation angioplasty of peripheral pulmonary stenosis associated with williams syndrome. Circulation.

[46] Helfrich AM, Philla KQ (2015). Late-onset hypercalcemia in Williams-Beuren syndrome: importance of early and frequent screening and intervention. J. Pediatr. Endocrinol. Metab..

[47] Dykens EM, Rosner BA, Ly T, Sagun J (2005). Music and anxiety in Williams syndrome: a harmonious or discordant relationship?. Am. J. Ment. Retard..

[48] Fritsch C, Bolsen K, Ruzicka T, Goerz G (1984). Congenital erythropoietic porphyria. Skin. Pharmacol. Phys..

[49] Lee WH, Tai WC, Wu PY (2012). Congenital erythropoietic porphyria. Dermatol. Sin..

[50] Di Pierro E, Brancaleoni V, Granata F (2016). Advances in understanding the pathogenesis of congenital erythropoietic porphyria. Br. J. Haematol..

[51] Poh-Fitzpatrick MB (1998). Clinical features of the porphyrias. Clin. Dermatol..

[52] Berry AA (2005). Two brothers with mild congenital erythropoietic porphyria due to a novel genotype. Arch. Dermatol..

[53] Pandey M (2013). Report of a novel Indian case of congenital erythropoietic porphyria and overview of therapeutic options. J. Pediatr. Hematol. Oncol..

[54] Poh-Fitzpatrick MB (1986). The erythropoietic porphyrias. Dermatol. Clin..

[55] Braun-Falco, O., Plewig, G., Wolff, H. H. & Burgdorf, W. H. C. *The Porphyrias* (Springer, Berlin, Heidelberg, 2000).

[56] Hallai N (2007). Pregnancy in a patient with congenital erythropoietic porphyria. N. Engl. J. Med..

[57] Takamura N (2002). Need for measurement of porphyrins in teardrops in patients with congenital erythropoietic porphyria. Br. J. Ophthalmol..

[58] Katugampola RP (2012). Congenital erythropoietic porphyria: a single-observer clinical study of 29 cases. Br. J. Dermatol..

[59] Fityan A, Fassihi H, Sarkany R (2016). Congenital erythropoietic porphyria: mild presentation with late onset associated with a mutation in the UROS gene promoter sequence. Clin. Exp. Dermatol..

[60] Darwich E (2011). Congenital erythropoietic porphyria and Parkinson's disease: clinical association in a patient with a long-term follow-up. Eur. J. Dermatol..

[61] Sidorsky TI, Christine CW, Epstein JH, Berger TG (2014). Development of corticobasal syndrome in a patient with congenital erythropoietic porphyria. Park. Relat. Disord..

[62] Harada FA, Shwayder TA, Desnick RJ, Lim HW (2001). Treatment of severe congenital erythropoietic porphyria by bone marrow transplantation. J. Am. Acad. Dermatol..

[63] Piomelli S, Poh-Fitzpatrick MB, Seaman C, Skolnick LM, Berdon WE (1986). Complete suppression of the symptoms of congenital erythropoietic porphyria by long-term treatment with high-level transfusions. N. Engl. J. Med..

[64] Peinado CM (2013). Successful treatment of congenital erythropoietic porphyria using matched unrelated hematopoietic stem cell transplantation. Pediatr. Dermatol..

[65] Murphy A, Gibson G, Elder GH, Otridge BA, Murphy GM (1995). Adult-onset congenital erythropoietic porphyria (Gunther's disease) presenting with thrombocytopenia. J. R. Soc. Med..

[66] Katugampola RP (2012). A management algorithm for congenital erythropoietic porphyria derived from a study of 29 cases. Br. J. Dermatol..

[67] Roughley PJ, Rauch F, Glorieux FH (2003). Osteogenesis imperfecta - clinical and molecular diversity. Eur. Cell. Mater..

[68] Marini, J. C. et al. Osteogenesis imperfecta. *Nat. Rev. Dis. Prim.* **3**, 17052 (2017). 10.1038/nrdp.2017.52 28820180

[69] Forlino A, Marini JC (2016). Osteogenesis imperfecta. Lancet.

[70] Rauch F, Glorieux FH (2004). Osteogenesis imperfecta. Lancet.

[71] Sillence DO, Senn A, Danks DM (1979). Genetic heterogeneity in osteogenesis imperfecta. J. Med. Genet..

[72] Brizola E, Staub ALP, Félix TM (2014). Muscle strength, joint range of motion, and gait in children and adolescents with osteogenesis imperfecta. Pediatr. Phys. Ther..

[73] Singer RB, Ogston SA, Paterson CR (2001). Mortality in various types of osteogenesis imperfecta. J. Insur. Med..

[74] Swinnen FKR (2011). Osteogenesis imperfecta: the audiological phenotype lacks correlation with the genotype. Orphanet J. Rare Dis..

[75] Land C, Rauch F, Montpetit K, Ruck-Gibis J, Glorieux FH (2006). Effect of intravenous pamidronate therapy on functional abilities and level of ambulation in children with osteogenesis imperfecta. J. Pediatr..

[76] Esposito P, Plotkin H (2008). Surgical treatment of osteogenesis imperfecta: current concepts. Curr. Opin. Pediatr..

[77] Krishnan H (2013). Primary and revision total hip arthroplasty in osteogenesis imperfecta. Hip Int..

[78] Topouchian V, Finidori G, Glorion C, Padovani JP, Pouliquen JC (2004). Posterior spinal fusion for kypho-scoliosis associated with osteogenesis imperfecta: long-term results. Rev. Chir. Orthop. Reparatrice. Appar. Mot..

[79] Janus GJM, Finidori G, Engelbert RHH, Pouliquen M, Pruijs JEH (2000). Operative treatment of severe scoliosis in osteogenesis imperfecta: results of 20 patients after halo traction and posterior spondylodesis with instrumentation. Eur. Spine J..

[80] McAllion SJ, Paterson CR (1996). Causes of death in osteogenesis imperfecta. J. Clin. Pathol..

[81] Falvo KA, Klain DB, Krauss AN, Root L, Auld PA (1973). Pulmonary function studies in osteogenesis imperfecta. Am. Rev. Respir. Dis..

[82] Carpenter TO, Imel EA, Holm IA, de Beur SMJ, Insogna KL (2011). A clinician's guide to X-linked hypophosphatemia. J. Bone Miner. Res..

[83] Lee JY, Imel EA (2013). The changing face of hypophosphatemic disorders in the FGF-23 era. Pediatr. Endocrinol. Rev..

[84] Pavone V (2015). Hypophosphatemic rickets: etiology, clinical features and treatment. Eur. J. Orthop. Surg. Traumatol..

[85] Kinoshita Y, Fukumoto S (2018). X-linked hypophosphatemia and FGF23-related hypophosphatemic diseases: prospect for new treatment. Endocr. Rev..

[86] Vakharia JD, Matlock K, Taylor HO, Backeljauw PF, Topor LS (2018). Craniosynostosis as the presenting feature of X-linked hypophosphatemic rickets. Pediatrics.

[87] Connor J (2015). Conventional therapy in adults with X-linked hypophosphatemia: effects on enthesopathy and dental disease. J. Clin. Endocrinol. Metab..

[88] Velan GJ, Currier BL, Clarke BL, Yaszemski MJ (2001). Ossification of the posterior longitudinal ligament in vitamin D-resistant rickets. Spine.

[89] Vega RA (2016). Hypophosphatemic rickets and craniosynostosis: a multicenter case series. J. Neurosurg. Pediatr..

[90] Souza MA, Junior LAVS, Santos MAD, Vaisbich MH (2010). Dental abnormalities and oral health in patients with Hypophosphatemic rickets. Clinics.

[91] Souza AP (2013). Dental manifestations of patient with Vitamin D-resistant rickets. J. Appl. Oral. Sci..

[92] Murayama T, Iwatsubo R, Akiyama S, Amano A, Morisaki I (2000). Familial hypophosphatemic vitamin D-resistant rickets: dental findings and histologic study of teeth. Oral. Surg. Oral. Med. Oral. Pathol. Oral. Radiol. Endod..

[93] Rabbani A, Rahmani P, Ziaee V, Ghodoosi S (2012). Dental problems in hypophosphatemic rickets, a cross sectional study. Iran. J. Pediatr..

[94] Soares ECS, Costa FWG, Ribeiro TR, Alves APNN, Fonteles CSR (2012). Clinical approach in familial hypophosphatemic rickets: report of three generations. Spec. Care. Dent..

[95] Sabandal MMI, Robotta P, Bürklein S, Schäfer E (2015). Review of the dental implications of X-linked hypophosphataemic rickets (XLHR). Clin. Oral. Investig..

[96] Andersen MG (2012). Periapical and endodontic status of permanent teeth in patients with hypophosphatemic rickets. J. Oral. Rehabil..

[97] Vital SO (2012). Tooth dentin defects reflect genetic disorders affecting bone mineralization. Bone.

[98] Chaussain-Miller C (2003). Dental abnormalities in patients with familial hypophosphatemic vitamin D-resistant rickets: prevention by early treatment with 1-hydroxyvitamin D. J. Pediatr..

[99] Batra P, Tejani Z, Mars M (2006). X-linked hypophosphatemia: dental and histologic findings. J. Can. Dent. Assoc..

[100] Douyere D, Joseph C, Gaucher C, Chaussain C, Courson F (2009). Familial hypophosphatemic vitamin D–resistant rickets—prevention of spontaneous dental abscesses on primary teeth: a case report. Oral. Surg. Oral. Med. Oral. Pathol. Oral. Radiol. Endod..

[101] Rathore R, Nalawade TM, Pateel D, Mallikarjuna R (2013). Oral manifestations of vitamin D resistant rickets in orthopantomogram. Case Rep..

[102] Capelli S (2015). Clinical and molecular heterogeneity in a large series of patients with hypophosphatemic rickets. Bone.

[103] Che H (2016). Impaired quality of life in adults with X-linked hypophosphatemia and skeletal symptoms. Eur. J. Endocrinol..

[104] Sharkey MS, Grunseich K, Carpenter TO (2015). Contemporary medical and surgical management of X-linked hypophosphatemic rickets. J. Am. Acad. Orthop. Surg..

[105] Mäkitie O (2008). Metabolic control and growth during exclusive growth hormone treatment in X-linked hypophosphatemic rickets. Horm. Res. Paediatr..

[106] Larson AN (2010). Hip and knee arthroplasty in hypophosphatemic rickets. J. Arthroplast..

[107] Carpenter TO (2018). Burosumab therapy in children with X-linked hypophosphatemia. N. Engl. J. Med..

[108] Carpenter TO (2014). Randomized trial of the anti-FGF23 antibody KRN23 in X-linked hypophosphatemia. J. Clin. Invest..

[109] Imel EA (2015). Prolonged correction of serum phosphorus in adults with X-linked hypophosphatemia using monthly doses of KRN23. J. Clin. Endocrinol. Metab..

[110] Linglart A, Biosse-Duplan M (2016). Hypophosphatasia. Curr. Osteoporos. Rep..

[111] Lia-Baldini A (2001). A molecular approach to dominance in hypophosphatasia. Hum. Genet..

[112] Millán JL (2013). The role of phosphatases in the initiation of skeletal mineralization. Calcif. Tissue Int..

[113] Millan JL, Plotkin H (2012). Hypophosphatasia - pathophysiology and treatment. Actual. Osteol..

[114] Silva I, Castelao W, Mateus M, Branco JC (2012). Childhood hypophosphatasia with myopathy: clinical report with recent update. Acta Reumatol. Port..

[115] Fraser (1957). D. Hypophosphatasia. Am. J. Med..

[116] Whyte MP (2015). Hypophosphatasia: validation and expansion of the clinical nosology for children from 25years experience with 173 pediatric patients. Bone.

[117] Whyte MP (2016). Hypophosphatasia — aetiology, nosology, pathogenesis, diagnosis and treatment. Nat. Rev. Endocrinol..

[118] Shohat M, Rimoin DL, Gruber HE, Lachman RS (1991). Perinatal lethal hypophosphatasia; clinical, radiologic and morphologic findings. Pediatr. Radiol..

[119] Silver MM, Vilos GA, Milne KJ (1988). Pulmonary hypoplasia in neonatal hypophosphatasia. Pediatr. Pathol..

[120] Whyte MP (2016). Asfotase alfa treatment improves survival for perinatal and infantile hypophosphatasia. J. Clin. Endocrinol. Metab..

[121] Bianchi ML (2015). Hypophosphatasia: an overview of the disease and its treatment. Osteoporos. Int..

[122] Whyte MP (2017). Hypophosphatasia: an overview for 2017. Bone.

[123] Hofmann C (2013). Clinical aspects of hypophosphatasia: an update. Clin. Rev. Bone Miner. Metab..

[124] Mornet E (2008). Hypophosphatasia. Best. Pract. Res. Clin. Rheumatol..

[125] Taketani T (2014). Clinical and genetic aspects of hypophosphatasia in Japanese patients. Arch. Dis. Child..

[126] Whyte MP (2010). Physiological role of alkaline phosphatase explored in hypophosphatasia. Ann. N. Y. Acad. Sci..

[127] Arun R, Khazim R, Webb JK, Burn J (2005). Scoliosis in association with infantile hypophosphatasia: a case study in two siblings. Spine.

[128] Sutton RAL, Mumm S, Coburn SP, Ericson KL, Whyte MP (2012). “Atypical femoral fractures” during bisphosphonate exposure in adult hypophosphatasia. J. Bone Miner. Res..

[129] Khandwala H, Mumm S, Whyte M (2006). Low serum alkaline phosphatase activity and pathologic fracture: case report and brief review of hypophosphatasia diagnosed in adulthood. Endocr. Pract..

[130] Coe JD, Murphy WA, Whyte MP (1986). Management of femoral fractures and pseudofractures in adult hypophosphatasia. J. Bone Jt. Surg..

[131] Guañabens N (2014). Calcific periarthritis as the only clinical manifestation of hypophosphatasia in middle-aged sisters. J. Bone Miner. Res..

[132] Beck C, Morbach H, Richl P, Stenzel M, Girschick HJ (2009). How can calcium pyrophosphate crystals induce inflammation in hypophosphatasia or chronic inflammatory joint diseases?. Rheumatol. Int..

[133] Rodriguez E (2012). Respiratory mechanics in an infant with perinatal lethal hypophosphatasia treated with human recombinant enzyme replacement therapy. Pediatr. Pulmonol..

[134] de Roo MGA (2014). Infantile hypophosphatasia without bone deformities presenting with severe pyridoxine-resistant seizures. Mol. Genet. Metab..

[135] Baumgartner-Sigl S (2007). Pyridoxine-responsive seizures as the first symptom of infantile hypophosphatasia caused by two novel missense mutations (c.677T>C, p.M226T; c.1112C>T, p.T371I) of the tissue-nonspecific alkaline phosphatase gene. Bone.

[136] Brumback RJ (1988). Intramedullary nailing of femoral shaft fractures. Part II: fracture-healing with static interlocking fixation. J. Bone Jt. Surg. Am..

[137] Whyte MP (2009). Chronic recurrent multifocal osteomyelitis mimicked in childhood hypophosphatasia. J. Bone Miner. Res..

[138] Deeb AA, Bruce SN, Morris AAM, Cheetham TD (2000). Infantile hypophosphatasia: disappointing results of treatment. Acta Paediatr..

[139] Taketani T (2015). *Ex vivo* expanded allogeneic mesenchymal stem cells with bone marrow transplantation improved osteogenesis in infants with severe hypophosphatasia. Cell Transplant..

[140] Whyte MP (2012). Enzyme-replacement therapy in life-threatening hypophosphatasia. N. Engl. J. Med..

[141] McKusick VA (1991). The defect in Marfan syndrome. Nature.

[142] Matt P (2008). Recent advances in understanding Marfan syndrome: should we now treat surgical patients with losartan?. J. Thorac. Cardiovasc. Surg..

[143] Ng CM (2004). TGF-β–dependent pathogenesis of mitral valve prolapse in a mouse model of Marfan syndrome. J. Clin. Invest..

[144] Pyeritz RE (2000). The marfan syndrome. Annu. Rev. Med..

[145] Loeys BL (2010). The revised Ghent nosology for the Marfan syndrome. J. Med. Genet..

[146] Lundby R (2011). CT of the hips in the investigation of protrusio acetabuli in Marfan syndrome. A case control study. Eur. Radiol..

[147] Lopes KRM (2006). Prenatal Marfan syndrome: report of one case and review of the literature. Prenat. Diagn..

[148] Nollen GJ (2002). Pulmonary artery root dilatation in Marfan syndrome: quantitative assessment of an unknown criterion. Heart.

[149] Waldmuller S (2007). Genetic testing in patients with aortic aneurysms/dissections: a novel genotype/phenotype correlation?. Eur. J. Cardiothorac. Surg..

[150] van Karnebeek CDM (2001). Natural history of cardiovascular manifestations in Marfan syndrome. Arch. Dis. Child..

[151] Zadeh N (2011). Ectopia lentis as the presenting and primary feature in Marfan syndrome. Am. J. Med. Genet. A..

[152] Nemet AY, Assia EI, Apple DJ, Barequet IS (2006). Current concepts of ocular manifestations in Marfan syndrome. Surv. Ophthalmol..

[153] Dwyer EM, Troncale F (1965). Spontaneous pneumothorax and pulmonary disease in the marfan syndrome. Report of two cases and review of the literature. Ann. Intern. Med..

[154] Judge DP, Dietz HC (2005). Marfan's syndrome. Lancet.

[155] Maron BJ (2004). Recommendations for physical activity and recreational sports participation for young patients with genetic cardiovascular diseases. Circulation.

[156] Engelfriet PM, Boersma E, Tijssen JG, Bouma BJ, Mulder BJ (2006). Beyond the root: dilatation of the distal aorta in Marfan's syndrome. Heart.

[157] Ladouceur M (2007). Effect of beta-blockade on ascending aortic dilatation in children with the marfan syndrome. Am. J. Cardiol..

[158] Detaint D (2010). Rationale and design of a randomized clinical trial (Marfan Sartan) of angiotensin II receptor blocker therapy versus placebo in individuals with Marfan syndrome. Arch. Cardiovasc. Dis..

[159] Cañadas V, Vilacosta I, Bruna I, Fuster V (2010). Marfan syndrome. Part 2: treatment and management of patients. Nat. Rev. Cardiol..

[160] Collins MT (2003). Thyroid carcinoma in the McCune-Albright syndrome: contributory role of activating Gsα mutations. J. Clin. Endocrinol. Metab..

[161] Dumitrescu, C. E. & Collins, M. T. McCune-Albright syndrome. *Orphanet. J. Rare. Dis*. **3**, 12 (2008). 10.1186/1750-1172-3-12 PMC2459161 18489744

[162] Chen, H. McCune-Albright Syndrome. (New York: Springer, 2017).

[163] Boyce AM, Florenzano P, de Castro LF & Collins MT. *Fibrous Dysplasia/McCune-Albright Syndrome.* (Seattle: Univ. Washington Press, 2018).

[164] Brillante B, Guthrie L, van Ryzin C (2015). McCune–Albright syndrome: an overview of clinical features. J. Pediatr. Nurs..

[165] Pichard DC, Boyce AM, Collins MT, Cowen EW (2014). Oral pigmentation in McCune-Albright syndrome. JAMA Dermatol..

[166] Collins MT, Singer FR, Eugster E (2012). McCune-Albright syndrome and the extraskeletal manifestations of fibrous dysplasia. Orphanet J. Rare Dis..

[167] Lee JS (2012). Clinical guidelines for the management of craniofacial fibrous dysplasia. Orphanet J. Rare Dis..

[168] Salenave S, Boyce AM, Collins MT, Chanson P (2014). Acromegaly and McCune-Albright syndrome. J. Clin. Endocrinol. Metab..

[169] Akintoye SO (2003). Dental characteristics of fibrous dysplasia and McCune-albright syndrome. Oral. Surg. Oral. Med. Oral. Pathol. Oral. Radiol. Endod..

[170] Akintoye SO, Boyce AM, Collins MT (2013). Dental perspectives in fibrous dysplasia and McCune–Albright syndrome. Oral. Surg. Oral. Med. Oral. Pathol. Oral. Radiol..

[171] Sadeghi SM, Hosseini SN (2011). Spontaneous conversion of fibrous dysplasia into osteosarcoma. J. Craniofac. Surg..

[172] de Araújo PIMP, Soares VYR, Queiroz AL, Santos AMD, Nascimento LA (2012). Sarcomatous transformation in the McCune–Albright syndrome. Oral. Maxillofac. Surg..

[173] Leopardi M, Parziale V, Ciavarella D, Chimenti C (2011). Maxillary bone lesions in McCune-Albright syndrome: a case report. Prog. Orthod..

[174] Akintoye SO (2002). Characterization ofgsp-mediated growth hormone excess in the context of McCune-Albright syndrome. J. Clin. Endocrinol. Metab..

[175] Robinson C, Collins MT, Boyce AM (2016). Fibrous dysplasia/McCune-Albright syndrome: clinical and translational perspectives. Curr. Osteoporos. Rep..

[176] Stanton RP (2012). The surgical management of fibrous dysplasia of bone. Orphanet J. Rare Dis..

[177] Paul SM (2014). Disease severity and functional factors associated with walking performance in polyostotic fibrous dysplasia. Bone.

[178] Becelli, R., Perugini, M., Cerulli, G., Carboni, A. & Renzi, G. Surgical treatment of fibrous dysplasia of the cranio-maxillo-facial area. Review of the literature and personal experience form 1984 to 1999. *Minerva Stomatol*. 51, 293–300 (2002). 12434124

[179] Kuznetsov SA (2008). Age-dependent demise of GNAS-mutated skeletal stem cells and “normalization” of fibrous dysplasia of bone. J. Bone Miner. Res..

[180] Riminucci M (1999). The histopathology of fibrous dysplasia of bone in patients with activating mutations of the Gs? Gene: site-specific patterns and recurrent histological hallmarks. J. Pathol..

[181] Ozawa T (2011). Long-term follow-up of a case of cheek hyperpigmentation associated with McCune-Albright syndrome treated with Q-switched ruby laser. Dermatol. Surg..

[182] Tessaris D (2012). Thyroid abnormalities in children and adolescents with McCune-Albright syndrome. Horm. Res. Paediatr..

[183] Stamou MI, Georgopoulos NA (2018). Kallmann syndrome: phenotype and genotype of hypogonadotropic hypogonadism. Metabolism.

[184] Balasubramanian, R. *Isolated Gonadotropin-Releasing Hormone (GnRH) Deficiency* (Seattle: Univ. Washington Press, 2017). 20301509

[185] Balasubramanian R, Crowley WF (2011). Isolated GnRH deficiency: a disease model serving as a unique prism into the systems biology of the GnRH neuronal network. Mol. Cell. Endocrinol..

[186] Mitchell AL, Dwyer A, Pitteloud N, Quinton R (2011). Genetic basis and variable phenotypic expression of Kallmann syndrome: towards a unifying theory. Trends Endocrinol. Metab..

[187] Boehm U (2015). European consensus statement on congenital hypogonadotropic hypogonadism—pathogenesis, diagnosis and treatment. Nat. Rev. Endocrinol..

[188] Tickotsky N, Moskovitz M (2014). Renal agenesis in kallmann syndrome: a network approach. Ann. Hum. Genet..

[189] Sato N (2004). Clinical assessment and mutation analysis of kallmann syndrome 1 (KAL1) and fibroblast growth factor receptor 1 (FGFR1, orKAL2) in five families and 18 sporadic patients. J. Clin. Endocrinol. Metab..

[190] Bailleul-Forestier I (2010). Dental agenesis in Kallmann syndrome individuals with FGFR1 mutations. Int. J. Paediatr. Dent..

[191] Nie M (2017). Analysis of genetic and clinical characteristics of a Chinese Kallmann syndrome cohort with ANOS1 mutations. Eur. J. Endocrinol..

[192] Aoyama K (2017). Molecular genetic and clinical delineation of 22 patients with congenital hypogonadotropic hypogonadism. J. Pediatr. Endocrinol. Metab..

[193] Laitinen EM, Hero M, Vaaralahti K, Tommiska J, Raivio T (2012). Bone mineral density, body composition and bone turnover in patients with congenital hypogonadotropic hypogonadism. Int. J. Androl..

[194] Iolascon G (2015). Bone involvement in males with Kallmann disease. Aging Clin. Exp. Res..

[195] Dodé C, Hardelin JP (2009). Kallmann syndrome. Eur. J. Hum. Genet..

[196] Chan E, Wayne C, Nasr A (2014). & FRCSC for Canadian Association of Pediatric Surgeon Evidence-Based Resource. Ideal timing of orchiopexy: a systematic review. Pediatr. Surg. Int..

[197] Levitus M (2005). The DNA helicase BRIP1 is defective in Fanconi anemia complementation group. J. Nat. Genet..

[198] Meetei AR (2003). A novel ubiquitin ligase is deficient in Fanconi anemia. Nat. Genet..

[199] Bagby GC, Alter BP (2006). Fanconi anemia. Semin. Hematol..

[200] Bagby GC (2003). Genetic basis of Fanconi anemia. Curr. Opin. Hematol..

[201] Godthelp BC, Artwert F, Joenje H, Zdzienicka MZ (2002). Impaired DNA damage-induced nuclear Rad51 foci formation uniquely characterizes Fanconi anemia group D1. Oncogene.

[202] Gowen LC, Avrutskaya AV, Latour AM, Koller BH, Leadon SA (1998). BRCA1 required for transcription-coupled repair of oxidative DNA damage. Science.

[203] Fagerlie SR, Koretsky T, Torok-Storb B, Bagby GC (2004). Impaired type I IFN-Induced Jak/STAT signaling in FA-C cells and abnormal CD4+ Th cell subsets in fancc-/- mice. J. Immunol..

[204] Pang Q (2001). Role of double-stranded RNA-dependent protein kinase in mediating hypersensitivity of Fanconi anemia complementation group C cells to interferon gamma, tumor necrosis factor-alpha, and double-stranded RNA. Blood.

[205] Chirnomas SD, Kupfer GM (2013). The inherited bone marrow failure syndromes. Pediatr. Clin. North Am..

[206] Rosenberg PS, Huang Y, Alter BP (2004). Individualized risks of first adverse events in patients with Fanconi anemia. Blood.

[207] Soulier J (2011). Fanconi anemia. Hematology.

[208] Sii-Felice K (2008). Role of fanconi DNA repair pathway in neural stem cell homeostasis. Cell Cycle.

[209] Alter BP, Rosenberg PS, Brody LC (2007). Clinical and molecular features associated with biallelic mutations in FANCD1/BRCA2. J. Med. Genet..

[210] Törnquist AL, Martin L, Winiarski J, Fahnehjelm KT (2014). Ocular manifestations and visual functions in patients with Fanconi anaemia. Acta Ophthalmol..

[211] Lin J, Kutler DI (2013). Why otolaryngologists need to be aware of Fanconi anemia. Otolaryngol. Clin. North. Am..

[212] Schneider M, Chandler K, Tischkowitz M, Meyer S (2015). Fanconi anaemia: genetics, molecular biology, and cancer-implications for clinical management in children and adults. Clin. Genet..

[213] de Latour RP (2015). Recommendations on hematopoietic stem cell transplantation for inherited bone marrow failure syndromes. Bone Marrow Transplant..

[214] MacMillan ML (2000). Haematopoietic cell transplantation in patients with Fanconi anaemia using alternate donors: results of a total body irradiation dose escalation trial. Br. J. Haematol..

[215] Kee Y, D’Andrea AD (2012). Molecular pathogenesis and clinical management of Fanconi anemia. J. Clin. Invest..

[216] Rio P (2014). Targeted gene therapy and cell reprogramming in Fanconi anemia. EMBO Mol. Med..

[217] Laimer M, Prodinger C, Bauer JW (2015). Hereditary epidermolysis bullosa. J. Dtsch. Dermatol. Ges..

[218] Intong LRA, Murrell DF (2012). Inherited epidermolysis bullosa: new diagnostic criteria and classification. Clin. Dermatol..

[219] Tabor A (2017). Raising awareness among healthcare providers about epidermolysis bullosa and advancing toward a cure. J. Clin. Aesthet. Dermatol..

[220] Has, C. & Fischer, J. Inherited epidermolysis bullosa: new diagnostics and new clinical phenotypes. *Exp. Dermatol*. 10.1111/exd.13668 (2018). 10.1111/exd.13668 29679399

[221] Fine, J. D. et al. The classification of inherited epidermolysis bullosa (EB): report of the third international consensus meeting on diagnosis and classification of EB. *J. Am. Acad. Dermatol*. **58**, 931–950 (2008). 10.1016/j.jaad.2008.02.004 18374450

[222] Haber RM, Hanna W, Ramsay CA, Boxall LBH (1985). Hereditary epidermolysis bullosa. J. Am. Acad. Dermatol..

[223] Mitsuhashi Y, Hashimoto I (2003). Genetic abnormalities and clinical classification of epidermolysis bullosa. Arch. Dermatol. Res..

[224] Wright JT, Fine JD, Johnson L (1993). Hereditary epidermolysis bullosa: oral manifestations and dental management. Pediatr. Dent..

[225] Gedde-Dahl, T. J. *Epidermolysis Bullosa: A Clinical, Genetic, and Epidemiologic Study* (Baltimore: John Hopkins Press, 1971).

[226] Wright JT, Fine JD, Johnson L (1994). Dental caries risk in hereditary epidermolysis bullosa. Pediatr. Dent..

[227] Kerns ML, DePianto D, Dinkova-Kostova AT, Talalay P, Coulombe PA (2007). Reprogramming of keratin biosynthesis by sulforaphane restores skin integrity in epidermolysis bullosa simplex. Proc. Natl Acad. Sci. USA.

[228] Cohn HI, Teng JMC (2016). Advancement in management of epidermolysis bullosa. Curr. Opin. Pediatr..

[229] Dormandy TL (1957). Gastrointestinal polyposis with mucocutaneous pigmentation (peutz–jeghers syndrome). N. Engl. J. Med..

[230] Tiainen M, Vaahtomeri K, Ylikorkala A, Makela TP (2002). Growth arrest by the LKB1 tumor suppressor: induction ofp21(WAF1/CIP1). Hum. Mol. Genet..

[231] Karuman P (2001). The peutz-jegher gene product LKB1 is a mediator of p53-dependent cell death. Mol. Cell.

[232] Morton DG, Roos JM, Kemphues KJ (1992). Par-4, a gene required for cytoplasmic localization and determination of specific cell types in *Caenorhabditis elegans* embryogenesis. Genetics.

[233] Beggs AD (2010). Peutz-Jeghers syndrome: a systematic review and recommendations for management. Gut.

[234] James, W. D., Elston, D. M. & Berger, T. G. *Andrews' Diseases of the Skin: Clinical Dermatology*, 7e. (Philadelphia: Elsevier, 2005).

[235] Choudhury, S., Das, A., Misra, P. & Ray, U. & Sarangi, S. Peutz-jeghers syndrome: a circumventable emergency. *Indian J. Dermatol.* 63, 168–171 (2018). 10.4103/ijd.IJD_563_16 PMC5903049 29692461

[236] Boardman LA (1998). Increased risk for cancer in patients with the peutz-jeghers syndrome. Ann. Intern. Med..

[237] Ryan DP, Hong TS, Bardeesy N (2014). Pancreatic adenocarcinoma. N. Engl. J. Med..

[238] Weng MT, Ni YH, Su YN, Wong JM, Wei SC (2010). Clinical and genetic analysis of peutz-jeghers syndrome patients in Taiwan. J. Formos. Med. Assoc..

[239] Choi HS, Park YJ, Park JG (1999). Peutz-Jeghers syndrome: a new understanding. J. Korean Med. Sci..

[240] Higham P, Alawi F, Stoopler ET (2010). Medical management update: peutz jeghers syndrome. Oral. Surg. Oral. Med. Oral. Pathol. Oral. Radiol. Endod..

[241] Tebani A (2016). Clinical and molecular characterization of patients with mucopolysaccharidosis type I in an algerian series. Int. J. Mol. Sci..

[242] Melbouci M (2018). Growth impairment in mucopolysaccharidoses. Mol. Genet. Metab..

[243] Neufeld, E. F. & Muenzer, J. in *The Metabolic and Molecular Bases of Inherited Disease* (eds Scriver, C. R., Beaudet, A. L., Sly, W. S. & Valle, D.) (New-York: McGraw-Hill, 2001).

[244] Różdżyńska-Świątkowska A, Jurecka A, Cieślik J, Tylki-Szymańska A (2015). Growth patterns in children with mucopolysaccharidosis I and II. World J. Pediatr..

[245] Martin R (2008). Recognition and diagnosis of mucopolysaccharidosis II (hunter syndrome). Pediatrics.

[246] de Ruijter J (2014). Growth in patients with mucopolysaccharidosis type III (Sanfilippo disease). J. Inherit. Metab. Dis..

[247] Montaño AM, Tomatsu S, Brusius A, Smith M, Orii T (2008). Growth charts for patients affected with Morquio A disease. Am. J. Med. Genet. A.

[248] Quartel A (2015). Growth charts for individuals with mucopolysaccharidosis VI (maroteaux-lamy syndrome). JIMD Rep..

[249] Montaño AM (2016). Clinical course of sly syndrome (mucopolysaccharidosis type VII). J. Med. Genet..

[250] Keilmann A, Bendel F, Nospes S, Lampe C, Läßig AK (2015). Alterations of mucosa of the larynx and hypopharynx in patients with mucopolysaccharidoses. J. Laryngol. Otol..

[251] Shapiro EG, Jones SA, Escolar ML (2017). Developmental and behavioral aspects of mucopolysaccharidoses with brain manifestations — neurological signs and symptoms. Mol. Genet. Metab..

[252] Sarmento DJdS (2018). Relationship between occlusal features and enzyme replacement therapy in patients with mucopolysaccharidoses. J. Oral. Maxillofac. Surg..

[253] de Almeida-Barros RQ (2012). Oral and systemic manifestations of mucopolysaccharidosis type VI: a report of seven cases. Quintessence Int..

[254] Kantaputra PN (2013). Oral manifestations of 17 patients affected with mucopolysaccharidosis type VI. J. Inherit. Metab. Dis..

[255] Alpöz AR (2006). The oral manifestations of Maroteaux-Lamy syndrome (mucopolysaccharidosis VI): a case report. Oral. Surg. Oral. Med. Oral. Pathol. Oral. Radiol. Endod..

[256] Giugliani R (2010). Mucopolysaccharidosis I, II, and VI: brief review and guidelines for treatment. Genet. Mol. Biol..

[257] Harmatz P (2010). Enzyme replacement therapy with galsulfase for mucopolysaccharidosis VI: clinical facts and figures. Turk. J. Pediatr..

[258] Valayannopoulos V, Wijburg FA (2011). Therapy for the mucopolysaccharidoses. Rheumatology.

[259] Muenzer J, Wraith JE, Clarke LA (2009). Mucopolysaccharidosis I: management and treatment guidelines. Pediatrics.

[260] Parini R (2017). Open issues in mucopolysaccharidosis type I-hurler. Orphanet J. Rare Dis..

[261] Kubaski F (2017). Hematopoietic stem cell transplantation for patients with mucopolysaccharidosis II. Biol. Blood. Marrow Transplant..

[262] Fan JQ (2008). A counterintuitive approach to treat enzyme deficiencies: use of enzyme inhibitors for restoring mutant enzyme activity. Biol. Chem..

[263] Piotrowska E (2011). Two-year follow-up of sanfilippo disease patients treated with a genistein-rich isoflavone extract: assessment of effects on cognitive functions and general status of patients. Med. Sci. Monit..

[264] Giugliani R, Harmatz P, Wraith JE (2007). Management guidelines for mucopolysaccharidosis VI. Pediatrics.

[265] Noh H, Lee JI (2014). Current and potential therapeutic strategies for mucopolysaccharidoses. J. Clin. Pharm. Ther..

[266] Sawamoto K, Chen HH, Almeciga-Diaz CJ, Mason RW, Tomatsu S (2018). Gene therapy for mucopolysaccharidoses. Mol. Genet. Metab..

[267] Mikulicz, J. Über Eine Eigenartige Symmetrische Erkrankung der Tranen und Mundspeicheldrusen (Stuttgart: Theodor Billroth, 1892).

[268] Yao Q, Wu G, Hoschar A (2013). IgG4-related Mikulicz's disease is a multiorgan lymphoproliferative disease distinct from Sjogren's syndrome: a Caucasian patient and literature review. Clin. Exp. Rheumatol..

[269] Umehara H (2012). Comprehensive diagnostic criteria for IgG4-related disease (IgG4-RD), 2011. Mod. Rheumatol..

[270] Maehara T (2012). Interleukin-21 contributes to germinal centre formation and immunoglobulin G4 production in IgG4-related dacryoadenitis and sialoadenitis, so-called Mikulicz's disease. Ann. Rheum. Dis..

[271] Bhatti RM, Stelow EB (2013). IgG4-related disease of the head and neck. Adv. Anat. Pathol..

[272] Yamamoto M, Takahashi H, Sugai S, Imai K (2005). Clinical and pathological characteristics of Mikulicz's disease (IgG4-related plasmacytic exocrinopathy). Autoimmun. Rev..

[273] Nelson WR, Kay S, Salley JJ (1963). Mikuliczʼs disease of the palate. Ann. Surg..

[274] Hamano H (2001). High serum IgG4 concentrations in patients with sclerosing pancreatitis. N. Engl. J. Med..

[275] Hamed G (2007). Inflammatory lesions of the lung, submandibular gland, bile duct and prostate in a patient with IgG4-associated multifocal systemic fibrosclerosis. Respirology.

[276] Kitagawa S (2005). Abundant IgG4-positive plasma cell infiltration characterizes chronic sclerosing sialadenitis (Kuttner’s tumor). Am. J. Surg. Pathol..

[277] Abe A (2014). The clinical characteristics of patients with IgG4-related disease with infiltration of the labial salivary gland by IgG4-positive cells. Mod. Rheumatol..

[278] Himi T, Takano K, Yamamoto M, Naishiro Y, Takahashi H (2012). A novel concept of Mikulicz's disease as IgG4-related disease. Auris Nasus Larynx.

[279] Moriyama M (2013). Clinical characteristics of Mikulicz’s disease as an IgG4-related disease. Clin. Oral. Investig..

[280] Merlini G, Bellotti V (2003). Molecular mechanisms of amyloidosis. N. Engl. J. Med..

[281] Borowicz J, Gillespie M, Miller R (2011). Cutaneous amyloidosis. Skinmed.

[282] Merlini G, Comenzo RL, Seldin DC, Wechalekar A, Gertz MA (2014). Immunoglobulin light chain amyloidosis. Expert. Rev. Hematol..

[283] Caccialanza R (2006). Nutritional status of outpatients with systemic immunoglobulin light-chain amyloidosis. Am. J. Clin. Nutr..

[284] Milani P, Merlini G, Palladini G (2018). Light chain amyloidosis. Mediterr. J. Hematol. Infect. Dis..

[285] Nelson LM, Gustafsson F, Gimsing P (2014). Characteristics and long-term outcome of patients with systemic immunoglobulin light-chain amyloidosis. Acta Haematol..

[286] Prokaeva T (2007). Soft tissue, joint, and bone manifestations of AL amyloidosis: clinical presentation, molecular features, and survival. Arthritis Rheum..

[287] Palladini G (2004). Association of melphalan and high-dose dexamethasone is effective and well tolerated in patients with AL (primary) amyloidosis who are ineligible for stem cell transplantation. Blood.

[288] Merlini G, Wechalekar AD, Palladini G (2013). Systemic light chain amyloidosis: an update for treating physicians. Blood.

[289] Gertz MA (2014). Immunoglobulin light chain amyloidosis: 2014 update on diagnosis, prognosis, and treatment. Am. J. Hematol..

[290] Clayton-Smith J, Laan L (2003). Angelman syndrome: a review of the clinical and genetic aspects. J. Med. Genet..

[291] Mabb AM, Judson MC, Zylka MJ, Philpot BD (2011). Angelman syndrome: insights into genomic imprinting and neurodevelopmental phenotypes. Trends Neurosci..

[292] Bird L (2014). Angelman syndrome: review of clinical and molecular aspects. Appl. Clin. Genet..

[293] Dagli A, Buiting K, Williams CA (2012). Molecular and clinical aspects of angelman syndrome. Mol. Syndromol..

[294] Pelc K, Boyd SG, Cheron G, Dan B (2008). Epilepsy in Angelman syndrome. Seizure.

[295] Williams CA, Driscoll DJ, Dagli AI (2010). Clinical and genetic aspects of Angelman syndrome. Genet. Med..

[296] Kyllerman, M. in *Handbook of Clinical Neurology* 3rd edn, Vol. 111 (eds Dulac, O., Lassonde, M. & Sarnat, H.) Ch. 32 (Philadelphia: Elseiver Press, 2013). 10.1016/B978-0-444-52891-9.09995-4 23622230

[297] Jaffe, R., Weiss, L. M. & Facchetti, F. WHO Classification of Tumours of Haematopoietic and Lymphoid Tissues. 4th edn, Vol. 2 (eds Swerdlow SH, Campo E, Harris NL, et al) Ch. 17 (Lyon, France: IARC. Press, 2008).

[298] Hand A (1893). Polyuria and tuberculosis. Arch. Pediatr..

[299] Broadbent V (1984). Spontaneous remission of multi-system histiocytosis X. Lancet.

[300] de Filippi P (2006). Specific polymorphisms of cytokine genes are associated with different risks to develop single-system or multi-system childhood Langerhans cell histiocytosis. Br. J. Haematol..

[301] Willman CL (1994). Langerhans'-cell histiocytosis (histiocytosis X)—a clonal proliferative disease. N. Engl. J. Med..

[302] Badalian-Very G (2010). Recurrent BRAF mutations in Langerhans cell histiocytosis. Blood.

[303] Rollins BJ (2015). Genomic alterations in langerhans cell histiocytosis. Hematol. Oncol. Clin. North. Am..

[304] Margo CE, Goldman DR (2008). Langerhans cell histiocytosis. Surv. Ophthalmol..

[305] Chu T (2001). Langerhans cell histiocytosis. Australas. J. Dermatol..

[306] Leonidas JC, Guelfguat M, Valderrama E (2003). Langerhans' cell histiocytosis. Lancet.

[307] Lian C, Lu Y, Shen S (2016). Langerhans cell histiocytosis in adults: a case report and review of the literature. Oncotarget.

[308] Meyer JS, de Camargo B (1998). The role of radiology in the diagnosis and follow-up of langerhans cell histiocytosis. Hematol. Oncol. Clin. North. Am..

[309] Kiratli H, Tarlan B, Söylemezoğlu F (2013). Langerhans cell histiocytosis of the orbit. Eur. J. Ophthalmol..

[310] Ginat DT, Johnson DN, Cipriani NA (2016). Langerhans cell histiocytosis of the temporal bone. Head. Neck Pathol..

[311] Zajko J (2013). Mandibular Langerhans cell histiocytosis in an adult. Bratisl. Lek. Listy.

[312] Coppes-Zantinga A, Egeler RM (2002). The Langerhans cell histiocytosis X files revealed. Br. J. Haematol..

[313] Krooks J, Minkov M, Weatherall AG (2018). Langerhans cell histiocytosis in children. J. Am. Acad. Dermatol..

[314] Gadner H (2008). Improved outcome in multisystem Langerhans cell histiocytosis is associated with therapy intensification. Blood.

[315] Gadner H (2013). Therapy prolongation improves outcome in multisystem Langerhans cell histiocytosis. Blood.

[316] Simko SJ, McClain KL, Allen CE (2015). Up-front therapy for LCH: is it time to test an alternative to vinblastine/prednisone?. Br. J. Haematol..

[317] Nagarajan R, Neglia J, Ramsay N, Baker KS (2001). Successful treatment of refractory langerhans cell histiocytosis with unrelated cord blood transplantation. J. Pediatr. Hematol. Oncol..

[318] Haroche J (2013). Dramatic efficacy of vemurafenib in both multisystemic and refractory Erdheim-Chester disease and Langerhans cell histiocytosis harboring the BRAF V600E mutation. Blood.

